# Intralayer/Interlayer Codoping Stabilizes Polarity Modulation in 2D Semiconductors for Scalable Electronics

**DOI:** 10.1002/advs.202408634

**Published:** 2024-10-24

**Authors:** Guitian Qiu, Lingan Kong, Mengjiao Han, Qian Zhang, Majeed Ur Rehman, Jianxian Yi, Lede Xian, Xiankai Lin, Aumber Abbas, Jiwei Chen, Yingjie Luo, Wenbo Li, Zhongchao Wei, Hongyun Meng, Xiuliang Ma, Qijie Liang

**Affiliations:** ^1^ School of Information and Optoelectronic Science and Engineering South China Normal University Guangzhou 510006 China; ^2^ Songshan Lake Materials Laboratory Dongguan 523808 China; ^3^ School of Materials Shenzhen Campus of Sun Yat‐sen University Shenzhen 518107 China

**Keywords:** CMOS devices, hole doping, intralayer/interlayer codoping, large‐area devices, superior air stability

## Abstract

2D semiconductors show promise as a competitive candidate for developing future integrated circuits due to their immunity to short‐channel effects and high carrier mobility at atomic layer thicknesses. The inherent defects and Fermi level pinning effect lead to n‐type transport characteristics in most 2D semiconductors, while unstable and unsustainable p‐type doping by various strategies hinders their application in many areas, such as complementary metal‐oxide‐semiconductor (CMOS) devices. In this study, an intralayer/interlayer codoping strategy is introduced that stabilizes p‐type doping in 2D semiconductors. By incorporating oppositely charged ions (F and Li) with the intralayer/interlayer of 2D semiconductors, remarkable p‐type doping in WSe_2_ and MoTe_2_ with air stability up to 9 months is achieved. Notably, the hole mobility presents a 100‐fold enhancement (0.7 to 92 cm^2^ V^−1^ s^−1^) with the codoping procedure. Structural and elemental characterizations, combined with theoretical calculations validate the codoping mechanism. Moreover, a CMOS inverter and more complex logic functions such as NOR and XNOR, as well as large‐area device arrays are demonstrated to showcase its applications and scalability. These findings suggest that stable and straightforward intralayer/interlayer codoping strategy with charge‐space synergy holds the key to unlocking the potential of 2D semiconductors in complex and scalable device applications.

## Graphical abstract

An intralayer/interlayer codoping strategy with charge‐space synergy is introduced to achieve stable and sustainable hole doping in 2D semiconductors. The hole mobility of WSe_2_ is enhanced by 100‐fold to 92 cm^2^ V^−1^ s^−1^. A complementary metal‐oxide‐semiconductor (CMOS) inverter and complex logic functions (NOR and XNOR), as well as large‐area device arrays are demonstrated to showcase its applications and scalability.

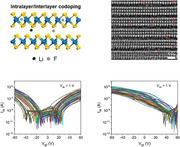

## 1 Introduction

2D semiconductors have garnered significant attention in the post‐Moore era, due to their atomically thin thickness, immunity to short‐channel effects, and high carrier mobility.^[^ ^1^, ^2^, ^3^ ^]^ As a promising candidate for electronic and optoelectronic devices, 2D semiconductors demonstrate immense potential in various applications, including light‐emitting diodes,^[^ ^4^, ^5^ ^]^ photodetectors,^[^ ^6^, ^7^ ^]^ solar cells,^[^ ^8^, ^9^ ^]^ and logic circuits.^[^ ^10^, ^11^ ^]^ Nonetheless, a pivotal challenge in the realm of 2D devices lies in achieving stable p‐type polarity, which is critical for developing 2D complementary metal‐oxide‐semiconductor (CMOS) logic circuits. The predominant n‐type or bipolar carrier transport observed in most 2D devices is largely attributed to intrinsic defects and the Fermi‐level pinning effect, which arises from metal‐induced gap states.^[^ ^12^ ^]^ Traditional channel doping techniques, such as high‐energy ion implantation, are inappropriate for delicate 2D lattices due to their aggressive kinetic energy. Therefore, achieving stable p‐type polarity in 2D semiconductors remains a significant hurdle for practical applications.^[^ ^13^, ^14^ ^]^ Addressing this challenge is crucial for unlocking the full potential of 2D semiconductors in advanced electronic and optoelectronic systems.

Numerous attempts have been made to effectively modulate the polarity of 2D semiconductors. Early research has primarily focused on selective doping methods, which are extensively studied strategies for controlling the polarity of various 2D semiconductors. These methods can be broadly categorized into element substitution doping and surface charge transfer doping (SCTD). For instance, the introduction of external atoms during chemical vapor deposition growth has been utilized to modulate the p‐type doping of different 2D semiconductors, such as MoS_2_, MoSe_2_, and WS_2_.^[^ ^15^, ^16^, ^17^ ^]^ Although this method offers simplicity in operation and can achieve the desired majority carrier type, it still suffers from issues such as channel lattice distortion and the introduction of defect states. Alternatively, SCTD presents a gentle physical/chemical surface absorption approach that avoids damaging the 2D lattice. Due to the difference in Fermi levels, the channel surface of 2D semiconductors and dopant materials generate a built‐in potential, leading to band bending and charge transfer. Consequently, p‐type doping can occur on 2D semiconductors when the Fermi level is higher than that of the dopant materials. The selective surface charge chemical doping of metal compounds offers the advantages of compatibility with the construction of 2D devices. This technique has been employed to successfully transform MoS_2_ from n‐type to robust p‐type transport, demonstrating its potential application in CMOS logic circuits.^[^ ^18^ ^]^ However, this SCTD strategy often falls short in terms of long‐term stability, which is primarily ascribed to the weak interaction and poor air stability of the dopants adsorbed on 2D semiconductor.^[^ ^19^ ^]^ To improve doping stability, it is crucial to carefully consider the native doping location and interface conditions. This is essential for fabricating high‐performance, large‐scale CMOS logic circuits. Recently, another approach has been demonstrated for polarity modulation in 2D SnS_2_ channels using a solvent‐based intercalation strategy. By inserting copper atoms into few‐layer SnS_2_, its natural n‐type behavior can be converted into p‐type transport. This intercalation doping method enables the construction of high‐performance SnS_2_ transistors and p‐n heterostructures.^[^ ^20^ ^]^ However, the choice of solvent is crucial for successful polarity modulation. This is because zerovalent metal intercalation in different solvents may lead to the formation of cation‐exchanged products. For instance, the use of copper in anhydrous methanol can transform layered SnS_2_ into a non‐layered Cu_2_SnS_3_ structure.^[^ ^21^ ^]^ Therefore, there is a pressing need to develop a p‐type modulation strategy that exhibits long‐term stability and scalability. This is essential for the realization of atomically thin integrated circuits and other functional devices.

In this work, we introduce an intralayer/interlayer codoping strategy that facilitates stable and scalable p‐type polarity modulation in 2D semiconductors. By using F and Li ions with two opposite charge states, which are distributed in the intralayer/interlayer of WSe_2_, a transition from bipolarity to predominantly hole transport is realized. This codoping is validated by density functional theory (DFT), high‐resolution transmission electron microscopy (HRTEM), and X‐ray photoelectron spectroscopy (XPS) combined with electron energy loss spectroscopy (EELS) characterizations. Remarkably, the p‐type doping with charge‐space synergy exhibits superior air stability of up to nine months, attesting to their outstanding stability. The Hole mobility is substantially enhanced by 100‐fold upon this codoping process. Furthermore, a CMOS inverter and more complex logic functions such as NOR and XNOR are demonstrated to showcase its advantages in device applications. We extend its application to MoTe_2_ and large‐area array WSe_2_ transistor devices, thereby not only validating the versatility of 2D semiconductor materials but also highlighting their potential for large‐scale integration. This work represents a significant advancement in the stable polarity modulation of 2D semiconductors, paving the way for their practical utilization in electronic applications.

## 2 Results and Discussions

**Figure 1:**
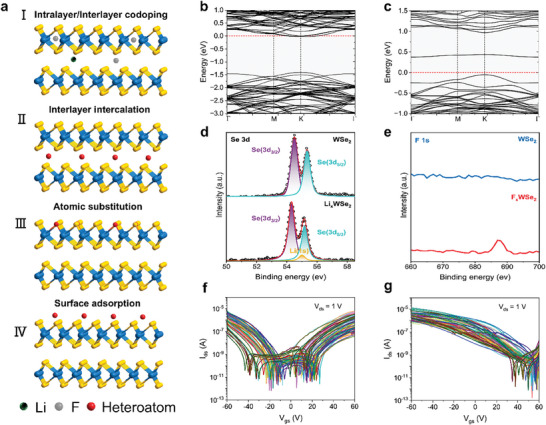
Intralayer/interlayer codoping in 2D semiconductors. a) Schematics diagram showing various doping strategies. b) Energy band structure of Li‐doped WSe_2_. c) Energy band structure of intralayer/interlayer codoped WSe_2_. XPS spectra of d) Li 1s peaks and e) F 1s peaks before (top) and after (bottom) doping. f,g) *I*_ds_–*V*_gs_ transfer characteristics of 60 pristine and codoped WSe_2_ samples.

Heteroatom doping has long been recognized as an effective method for hole doping in 2D semiconductors, encompassing techniques such as interlayer intercalation,^[^ ^20^ ^]^ atom substitution,^[^ ^22^, ^23^ ^]^ and surface adsorption.^[^ ^24^, ^25^ ^]^ However, the stability and sustainability of these approaches remain a significant hurdle for practical applications. To address this challenge, we introduce an intralayer/interlayer codoping strategy that leverages the opposing charge states of two distinct ions and their spatial distribution in intralayer and interlayer. This approach promises to stabilize hole doping in 2D semiconductors, as schematically depicted in **Figure** **1**a. Specifically, we employ a LiPF_6_ solution as a source of F and Li ions to facilitate p‐type doping in 2D WSe_2_. To gain deeper insights, DFT calculations were conducted to investigate the doping effects of F and Li ions. Individual Li ion doping tends to shift the Fermi level towards the conduction band, resulting in electron doping (Figures 1b and S1a, Supporting Information). Conversely, F ion doping exhibits a hole doping effect (Figure S1b,c, Supporting Information). When implementing the intralayer/interlayer codoping of F and Li ions (Figures 1c and S1d–f, Supporting Information), with F ions dominating the composition, a pronounced p‐type doping effect will be observed. Furthermore, we compared the internal stability of the dopants through formation energy calculations, as shown in Figure S2 (Supporting Information). The calculation results indicate that the intralayer/interlayer codoped system has the lowest formation energy, revealing the highest stability.

In the LiPF_6_ solution, a series of hydrolysis reactions occur, as shown in the following processes:^[^ ^26^, ^27^ ^]^

(1) $$\;\mathrm{LiP}F_{6}↔\mathrm{LiF}+PF_{5}$$

(2) $$\;PF_{5}+H_{2}O\to \mathrm{PO}F_{3}+2\mathrm{HF}$$

(3) $$\;\mathrm{PO}F_{3}+H_{2}O\to \mathrm{HP}O_{2}F_{2}+\mathrm{HF}$$

(4) $$\;\mathrm{HP}O_{2}F_{2}+H_{2}O\to H_{2}PO_{3}F+\mathrm{HF}$$

(5) $$\;H_{2}PO_{3}F+H_{2}O\to H_{3}PO_{4}+\mathrm{HF}$$

Consequently, the PF_6_^−^ in the solution produces abundant F ions, leading to a noticeable increase in the concentration of F⁻ during doping compared to that of Li^+^. To elucidate the compositional changes in multilayer WSe_2_ following codoping with LiPF_6_ solution, we conducted XPS analysis. As depicted in Figure 1d,e, the emergence of Li 1s and F 1s peaks at 55 eV and 687 eV, respectively, in the doped samples, in contrast to their absence in the pristine WSe_2_, signifies the successful incorporation of Li and F ions. The Se 3d and W 4f spectra exhibited a shift towards lower binding energies (Figure S3a,b, Supporting Information), indicative of a downward movement of the Fermi level towards the valence band edge, thereby facilitating p‐type doping.^[^ ^28^ ^]^ Furthermore, Raman spectroscopy analysis revealed a pronounced shift of the Raman peak towards higher wavenumbers after codoping (Figure S3c, Supporting Information). This observed shift is rationalized by the p‐type doping‐induced Fermi level displacement towards the valence band, which diminishes the electron concentration and consequently attenuates electron–phonon scattering, resulting in the observed blue shift of the Raman peak, in alignment with findings from prior studies.^[^ ^28^, ^29^ ^]^ Moreover, we measured the photoluminescence (PL) spectra by using the WSe_2_ thickness of 0.81 nm (Figure S3d,e, Supporting Information). With this thickness, we confirm the peak at 1.63 eV for pristine WSe_2_, which we further deconvoluted into neutral exciton emission at 1.64 eV and trion emission at 1.59 eV. The neutral excitons dominate the PL emission of the pristine WSe_2_. The PL peak of the WSe_2_ treated with a LiPF_6_ solution exhibits a blue shift to 1.64 eV compared to the pristine WSe_2_. Then, we deconvoluted this PL peak into neutral exciton emission at 1.65 eV and trion emission at 1.60 eV. Although the intensity of the trion increases and that of the exciton decreases after doping, the recombination of exciton still dominates in doped WSe_2_, resulting in the blue shift of the overall PL peak (Figure S3f, Supporting Information), which is also observed in other similar p‐type doped WSe_2_ and WS_2_.^[^ ^28^, ^30^ ^]^ Meanwhile, we found that the PL intensity of doped WSe_2_ was significantly reduced, as shown in Figure S3g (Supporting Information). This phenomenon can be attributed to the increased density of trions after doping, which leads to stronger electrostatic interactions between charged trions and the dopant impurities. As a result, this non‐radiative recombination would reduce the PL intensity of the doped sample. This is similar with previous reports on p‐type doping in 2D semiconductors.^[^ ^23^, ^31^ ^]^ These observations are attributed to the enhanced hole concentration resulting from p‐type doping.

To further elucidate the impact of doping ions on the electron concentration of WSe_2_, scanning Kelvin probe microscopy tests were conducted (see Experimental Section for details). The contact potential difference (V_CPD_), which reflects the variation in the sample's work function, is delineated by the equation:

(6) $$V_{\mathrm{CPD}}=\frac{W_{\mathrm{sample}\;}-\;W_{\mathrm{tip}}}{e}$$

where *W*_sample_ and *W*_tip_ are the work functions of the sample and the probe tip, and *e* is the elementary charge.^[^ ^32^ ^]^ As shown in Figure S4a (Supporting Information), a significant difference in surface potentials is observed between the pristine and doped WSe_2_. Specifically, the surface potential of the doped WSe_2_ increases by 41 mV compared to the pristine sample (Figure S4b, Supporting Information), indicating that the doping ions elevate the work function of WSe_2_ and concurrently reduce its electron concentration. We have conducted additional experiments to exclude the possible influence of photoresist on doping effect of 2D WSe_2_ (Figures S5–S7, Supporting Information).

According to the results of theoretical calculations, the codoped system with F as the dominant ion has stable p‐type doping effects. Therefore, we fabricated 60 WSe_2_ transistors with similar thickness (atomic force microscopy images in Figure S8, Supporting Information) to test the p‐type doping effect of the solution. Figure 1f,g presents the transfer curves of 60 samples before and after doping, respectively, demonstrating a remarkable transition from ambipolar transport to hole‐dominated transport with a 100% yield. This shows the advantages of the codoping strategy in effectiveness, reproducibility, and scalability.

**Figure 2:**
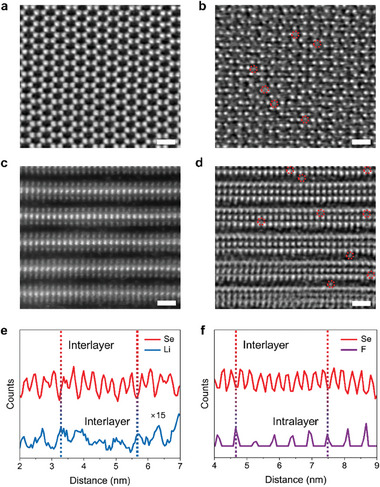
Structural and elemental characterizations verifying intralayer/interlayer codoping. a) iDPC‐STEM image of the pristine WSe_2_ flake. b) iDPC‐STEM image of the codoped WSe_2_ flake. Dopants are marked by dashed red circles. c) Cross‐sectional iDPC‐STEM images of the c) pristine and d) codoped WSe_2_ flakes. Doped Li and F ions are marked by dashed red circles in (d). Scale bar: 1 nm. e,f) EELS intensity line profiles of Se, Li and F, where F ions present in intralayer and interlayer and Li ions exist only in interlayer.

To confirm the presence of F and Li ions within the intralayer/interlayer of WSe_2_, as well as the distribution of these elements before and after codoping, HRTEM and EELS characterizations were conducted. Given the superior contrast and signal‐to‐noise ratio of the integrated differential phase contrast scanning transmission electron microscopy (iDPC‐STEM), it is crucial for our work to identify the light elements, particularly F and Li ions. **Figure** **2**a characterizes the pristine WSe_2_ surface image of iDPC‐STEM, exhibiting a highly ordered atomic arrangement with negligible defects and a clearly discernible 2H‐phase atomic structure with hexagonal symmetry.^[^ ^33^ ^]^ By contrast, the high angle annular dark field scanning transmission electron microscopy (HAADF‐STEM) image shows remarkably weaker signals and thus lower contrast (Figure S9a, Supporting Information). After codoping, iDPC‐STEM image reveals the presence of numerous dissociative ions, exhibiting diverse orientations within the WSe_2_ hexagonal lattice (marked red circles), ruling out the possibility of imaging artifact, as shown in Figure 2b. These ions cannot be directly identified by HAADF‐STEM, because F and Li ions have low electron scattering, resulting in the difficulty of collecting signals (Figure S9b, Supporting Information). Figure 2c,d further corroborates the existence of F and Li ions in WSe_2_ intralayer/interlayer, the cross‐section iDPC‐STEM image clearly demonstrates the emergence of new elements at different positions within WSe_2_ intralayer and interlayer (marked red circles), consistent with theoretical calculations and the observation of two light elements of Li and F ions (Figure 1b–e). It should be noted that these dopant ions do not induce any changes in the interlayer spacing of WSe_2_, as demonstrated by X‐ray diffraction (Figure S10, Supporting Information), which is consistent with the measurement of WSe_2_ interlayer spacing of 0.65 nm (Figures 2c and S11, Supporting Information).

In addition, EELS measurements offer the capability to ascertain the composition of intralayer and interlayer doping elements. Figure 2e shows the peak of Li is located between two Se peaks, separated by approximately 0.3 nm, indicating that Li ions are exclusively confined to the interlayer of the doped WSe_2_. In Figure 2f, the peak of F is located between the peaks of two Se at distances of 0.28 nm and 0.35 nm, respectively, indicating that F ions are present both within the intralayer and interlayer of the doped WSe_2_. Additionally, from our DFT calculations, we found that Li ions are impossible to be located at the intralayer sites. Even if we put a Li ion at the intralayer site initially, it will move to the interlayer sites after structural relaxation. Therefore, following the doping process, Li ions are confined to the interlayer of WSe_2_, which is consistent with the results of EELS. According to theoretical calculations, F ion doped WSe_2_ reveals hole dominated p‐type behavior, while Li‐doped WSe_2_ exhibits electron‐dominated n‐type characteristics. It is obvious that the concentration of F ions in doped WSe_2_ is significantly higher than that of Li ions, leading to the overall dominance of p‐type behavior in WSe_2_.

**Figure 3:**
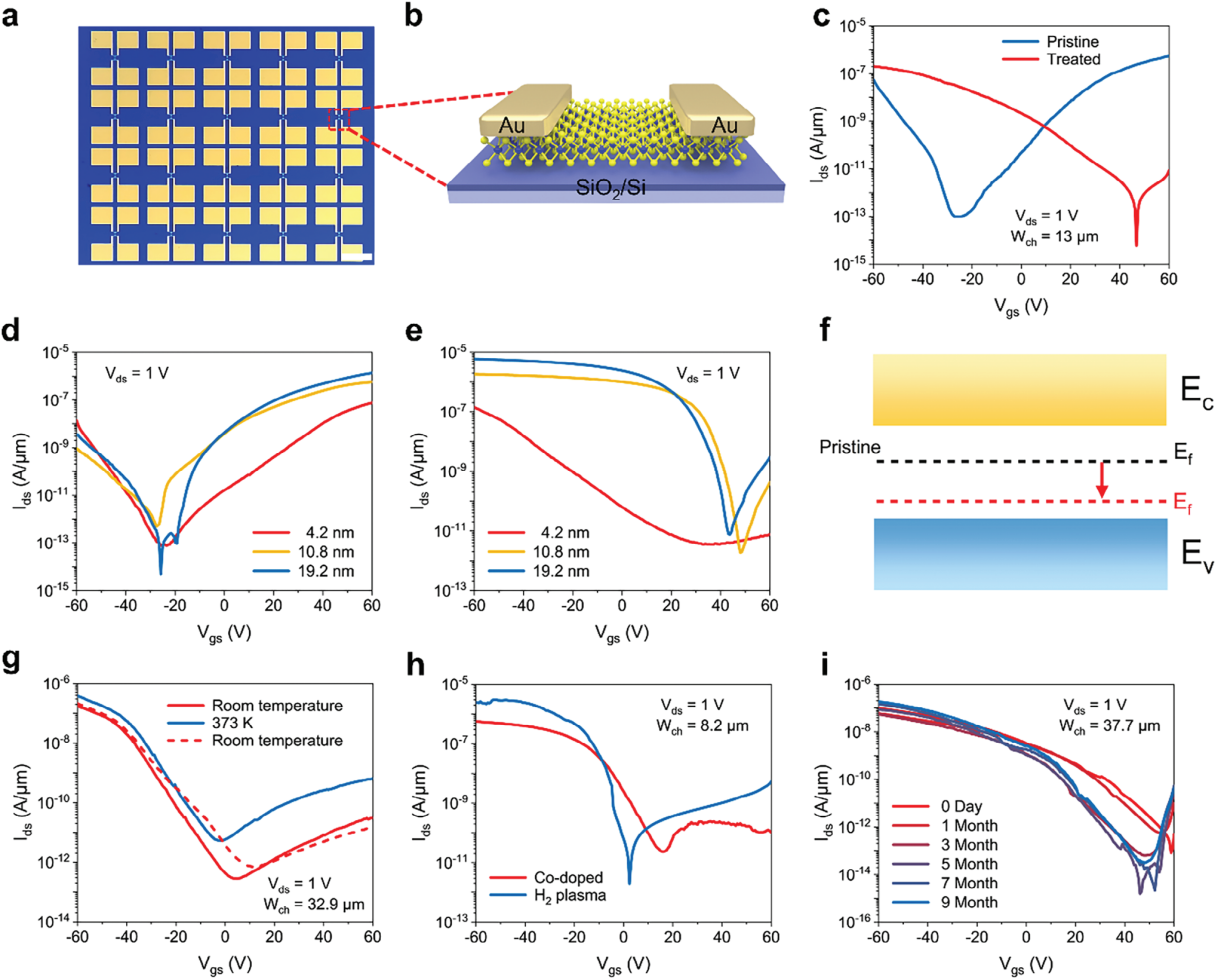
Electrical transport properties illustrating the feasibility and stability of the intralayer/interlayer codoping strategy. a) Optical image of the WSe_2_ device arrays. Scale bar: 1 mm. b) Schematic of the individual transistor. c) *I*_ds_–*V*_gs_ transfer curves of the pristine and codoped WSe_2_ transistors. d,e) Thickness‐dependent transfer characteristics of the pristine and codoped WSe_2_ transistors. The channel width (*W*_ch_) is 13.1, 20.1, and 20.0 nm, respectively. f) Schematic of the change of the energy band structure. g) Temperature‐dependent transfer characteristics of codoped WSe_2_ transistors. Red dashed line indicates transfer characteristics after returning to room temperature. h) Doping stability against mild H_2_ plasma. i) Air‐stability of codoped WSe_2_.

To systematically investigate the effectiveness and stability of codoping strategy on 2D semiconductors, we fabricated back‐gated transistors utilizing multilayer WSe_2_. **Figure** **3**a shows the optical image of the 4 × 5 device arrays in which the channel of 3.2 nm thick WSe_2_ films (Figure S12, Supporting Information) and the schematic structure of individual device was shown in Figure 3b. The output curves for both the undoped and doped devices exhibit nonlinearity, indicating the Schottky contact exists between semiconductors and electrodes (Figure S13a,b, Supporting Information). The *I*_ds_–*V*_gs_ transfer curves of the pristine and codoped WSe_2_ FET in Figure 3c display prominent transition from ambipolar to p‐type dominated transport characteristics after doping for 5 h (see Experimental Section for details). The transfer curves applying different drain voltages at ‐60 V to 60 V back‐gate voltages give similar results (Figure S13c,d, Supporting Information).

The codoping strategy is applicable for 2D semiconductors with different thicknesses (Figure S14, Supporting Information). As shown in Figure 3d,e, the transfer curves display similar transformation from ambipolar to p‐type transport with thicknesses of WSe_2_ varied from 4.2 to 19.2 nm. From the analysis of DFT calculation results, the co‐insertion of F and Li ions will lead to the change of the band structure of WSe_2_. Consequently, the Fermi level shifts towards the valence band, resulting in p‐type doping of WSe_2_, as shown in Figure 3f. The carrier mobility is calculated by the following equation:

(7) $$\mu =\frac{dI_{\mathrm{ds}}}{dV_{\mathrm{gs}}}\;\frac{L}{W}\frac{1}{C}\;\frac{1}{V_{\mathrm{ds}}}$$

where *L* and *W* are the channel length and width, respectively, *C* (1.15 × 10^8^ F cm^−2^) is the capacitance of 300 nm SiO_2_, *V*_ds_ (1 V) is the drain voltage. For the undoped WSe_2_ FETs with thickness of 19.2 nm, the hole mobility of 0.7 cm^2^ V^−1^ s^−1^ is achieved. After codoping, this value significantly increases to 92 cm^2^ V^−1^ s^−1^. After codoping treatment, the hole mobility of WSe_2_ was increased by over 100‐fold. We attribute that this enhancement is mainly due to two aspects. First, the contact resistance (*R*_C_) of the device is significantly reduced after LiPF_6_ codoping. We use transfer length method (TLM) to measure the *R*_C_, which is reduced by three orders of magnitude after codoping (Figure S15, Supporting Information). Second, the process of codoping with F and Li ions represents a spontaneous intercalation that can enhance the carrier density of WSe_2_ without altering the number of scattering centers.^[^ ^20^, ^34^ ^]^ Therefore, the screening effect can reduce the effective scattering of carriers, resulting in an increased hole mobility in doped WSe_2_. In addition, we delved into the effect of different treating times on the doping effect (Figure S16, Supporting Information). The extended doping time by 5 h can produce decent modulation of the hole concentration. As the doping time increases, the distribution range of F and Li ions in WSe_2_ continues to expand, but their proportion remains constant, thereby enhancing the p‐type doping of WSe_2_. We fabricated WSe_2_ samples with similar thicknesses to conduct KPFM tests at different doping times (Figures S17 and S18, Supporting Information). After immersing the samples in LiPF_6_ solution for 30 minutes, 1 h, and 3 h, respectively, their surface potential difference exhibited a gradual increase, and the Fermi level gradually shifted towards the valence band. It indicates that the increase in F and Li ions distribution can alter the Fermi level and hole concentration of doped WSe_2_. Furthermore, the intralayer/interlayer codoping strategy has proven effective for 2D MoTe_2_ with a thickness of 9.5 nm (Figure S19a,b, Supporting Information), as shown in the transfer curves of the pristine and doped samples (Figure S19c,d, Supporting Information). These results illustrate the feasibility and advantages of the intralayer/interlayer codoping strategy.

The intralayer/interlayer codoping by ions with charge‐space synergy possesses great advantages in terms of stability and sustainability. The stability was thoroughly examined through the electrical transport properties evolution of WSe_2_ FETs in air, across different temperatures, and even following plasma bombardment. Notably, the doping effect persisted despite the temperature rising from room temperature to 373 K and then back, evident in the transfer curves of doped WSe_2_ FET in Figure 3g. The device thickness utilized for temperature stability testing is 12.5 nm (Figure S20, Supporting Information). Furthermore, the doping effect also exhibited some immunity to mild plasma bombardment. After using H_2_ plasma treat the codoped WSe_2_ and reduce its thickness by 0.9 nm (Figure S21, Supporting Information), the hole dominated transport maintained intact (Figure 3h). To eliminate any confounding factors, we replicated this H_2_ plasma treatment on undoped WSe_2_ device with a thickness of 5.7 nm (Figure S22a,b, Supporting Information), ruling out the possibility of hole doping solely attributed to H_2_ (Figure S22c, Supporting Information).

For practical applications, the long‐term stability of p‐type doped devices under environmental conditions is paramount. Remarkably, the codoped WSe_2_ device with a thickness of 8.1 nm (Figure S23, Supporting Information) maintained the hole doping effect for over 9 months, even without any protective layer, as shown in Figure 3i. The mechanism may be that Li and F ions act as n‐type and p‐type dopants respectively, while the Columbic attraction between these oppositely charged dopants poses a significant challenge to the removal of F ions from the WSe_2_ lattice.^[^ ^35^ ^]^ Therefore, compared to other polarity modulation strategies, such as atom substitution and surface adsorption, the intralayer/interlayer codoping presents remarkable stability and sustainability, highlighting its great potential in polarity modulation of 2D semiconductors. As summarized in **Table** **1**, we compared the p‐type doping strategies of 2D semiconductors in terms of hole mobility, air‐stability, and device demonstration,^[^ ^20^, ^22^, ^28^, ^29^, ^36^, ^37^, ^38^, ^39^, ^40^, ^42^ ^]^ the intralayer/interlayer codoping strategy with charge‐space synergy shows its comprehensive advantages in these aspects.

**Table 1:** Comparison of p‐type doping strategies for 2D materials.

Strategy	Materials	Dopants	Hole mobility [cm^2^ V^−1^s^−1^]	Air stability	Device applications	Scalability demonstration	Refs.
Substitutional doping	PdSe_2_	Sn	10	20 d	Photodetector	NA	[29]
	MoSe_2_	Ta	2.6	NA	Photodetector	NA	[22]
	MoS_2_	Nb	1.5	7 d	NA	Yes	[36]
	WS_2_	N	18.8	45 d	NA	Yes	[37]
SCTD	WSe_2_	Br_2_	27	NA	CMOS inverter	Yes	[42]
	MoTe_2_	O_2_	41	3 months	Lateral p–n junction	NA	[38]
	WSe_2_	4‐NBD	82	NA	CMOS inverter	NA	[28]
	WSe_2_	OTS	168	6 h	Photodetector	NA	[39]
	PtSe_2_	MMA/PMMA	14	NA	CMOS inverter	Yes	[40]
Intercalation doping	SnS_2_	Cu	40	NA	Lateral p‐n‐metal junction	NA	[20]
Intralayer/interlayer codoping	WSe_2_	F/Li	92	9 months	CMOS inverter logic gates	Yes	This work

**Figure 4:**
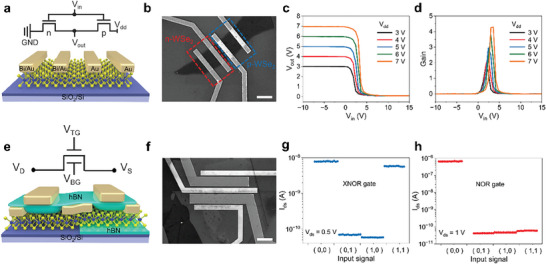
CMOS inverter and logic functions based on codoped WSe_2_ transistors. a) Circuit diagram (upper) and structure diagram (lower) of the inverter. b) SEM image of the inverter, where n‐type is marked by red dashed box, p‐type is marked by blue dashed box. Scale bar: 20 µm. c) Voltage transfer characteristics of inverter as a function of the input voltage with different *V*_dd_ from 3 to 7 V. d) Voltage gains of the inverter. e) Circuit diagram (upper) and structure diagram (lower) of NOR and XNOR logic gates. f) SEM image of the logic circuit. Scale bar: 20 µm. g,h) The input–output logic functions of g) XNOR and h) NOR.

With the ability to control p‐type branch of WSe_2_ transistor, we can readily fabricate CMOS functional circuits. For example, a complementary logic inverter can be effortlessly created by connecting two WSe_2_ transistors in series on a single flake with a thickness of 14.2 nm (Figure S24a,b, Supporting Information), where n‐type transport fabricated through the deposition of bismuth metal with a low work function, while the other is achieved through our codoping strategy combined with high work function of gold. By using metal contacts with different work functions, it is possible to effectively increase the injection of carriers at the contact interface (Figure S24c, Supporting Information). **Figure** **4**a,b, respectively, shows the logic diagram, schematic structure and scanning electron microscope (SEM) image of CMOS inverter. The corresponding *I*_ds_–*V*_gs_ transfer curves of individual electrical measurement are also provided (Figure S24d, Supporting Information). Thanks to the comparable drain current levels of both transistors, high‐performance of the voltage transfer characteristics of the inverter is plotted. With improving the various bias voltages, the voltage gains observably increase, a voltage transition with a maximum gain of 4 under applied voltage of 7 V is obtained, as shown in Figure 4c,d. Owing to these two transistors connected in series, that is, the carriers transport occurs the channel of between source and drain. Taking p‐type device as an example, Au metal contact to doped WSe_2_ branch is beneficial to hole carriers transport due to lower Schottky barrier height than Bi metal contact even though both metals are connected. Similarly, Bi metal contact takes precedence over Au contact for n‐type device. This CMOS inverter, fabricated on 300 nm thick SiO_2_ dielectric layer, holds promise for even higher voltage gains through using high‐*k* dielectric materials (such as Al_2_O_3_, HfO_2_) to enhance the gate coupling.^[^ ^41^, ^42^ ^]^

Make a step forward, more intricate logic circuits such as NOR and XNOR gates can be achieved by cleverly combining hBN and SiO_2_ to construct both the top and bottom dielectrics. The gate voltages are used as two input signals that can be applied four input states (0,0), (0,1), (1,0), (1,1), respectively, where 0 represents negative voltage and 1 stands for positive voltage. The schematic representation and SEM image of the device are shown in Figure 4e,f. Notably, one side of the transistor incorporates an additional 20 nm thick BN layer sandwiched between 6.5 nm thick WSe_2_ (Figure S25a,b, Supporting Information) and the SiO_2_ substrate, enhancing ambipolar transport capabilities (Figure S25c, Supporting Information).^[^ ^43^ ^]^ In an ambipolar transistor, when the input signal is (0,0) and (1,1), both electrons and holes can accumulate through the gate, resulting in a high drain current and an output signal of 1. When the input signal is (1,0) and (0,1), the changes in carrier concentration caused by the opposite polarities of the two gate voltages can be ignored, and the output signal is 0. This transistor corresponds to an XNOR gate, as shown in Figure 4g. The other side maintains p‐type transport after ions codoping (Figure S25d, Supporting Information). In a p‐type transistor, the carriers in the channel will accumulate and the drain current will be greatly enhanced only when both the top and bottom gate outputs are negative. When the input signal is (1,0) and (0,1), the charge carriers in the channel are accumulated by one gate and depleted by the other, resulting in an output signal of 0. This device works as NOR gate, as shown in Figure 4h. By meticulously controlling the WSe_2_ polarity, the NOR gate and XNOR gate are demonstrated with inputting different logic states,^[^ ^44^ ^]^ suggesting the potential for more complex logic functions. These demonstrations highlight the immense potential of devices employing the codoping strategy in the realm of 2D electronic devices, paving the way for even more sophisticated logic functions.

## 3 Conclusion

In summary, we introduce an intralayer/interlayer codoping approach utilizing ions with complementary charge states to achieve stable and sustainable p‐type doping in 2D semiconductors. Our strategy, involving the codoping with F and Li ions, has significantly enhanced the p‐type doping stability of 2D WSe_2_, maintaining performance over a 9 month period. Moreover, these devices exhibit robust stability across a range of temperatures and under mild plasma exposure. Remarkably, the hole mobility in the WSe_2_ FETs has been dramatically increased from an initial 0.7 to 92 cm^2^ V^−1^ s^−1^ through this codoping strategy. The atomic‐resolution structural analysis, elemental composition characterizations, and DFT calculations corroborate the intralayer/interlayer codoping. Furthermore, the versatility of this codoping is demonstrated through its successful application in other semiconductors, such as 2D MoTe_2_, while its scalability is confirmed through the fabrication of large‐area device arrays. Finally, the practical implications of this codoping method with charge‐space synergy are highlighted by the development of CMOS inverters and NOR/XNOR logic gates, showcasing its potential for device applications. These findings represent a significant step forward in stabilizing polarity modulation in 2D semiconductors, opening avenues for future advancements in 2D electronics and optoelectronics.

## 4 Experimental Section

### Density Functional Theory Calculation

The first‐principles DFT calculations were performed using the Vienna ab initio simulation package (VASP). The projector‐augmented‐wave (PAW) pseudopotentials approach was used to describe the interaction between atomic cores and valence electrons. The electron‐electron exchange‐correlation effects were treated using the generalized gradient approximation (GGA) in the Perdew‐Burke‐Ernzerhof (PBE) form. The many‐body wave functions were expanded in a large plane‐wave basis sets with an energy cutoff of 500 eV. A sufficiently thick vacuum buffer space‐layer of more than 25 Å was used to suppress the interaction between adjacent slabs. The supercell method was adopted to simulate the F and Li ions codoping effects. A large supercell of 4 × 4 × 1 (comprised of 16 primitive unit cells of WSe_2_) was constructed from a converged primitive unit cell of WSe_2_ to sufficiently minimize the possible interaction among the periodic images of the dopants. For the integration over the k‐space, a dense Γ‐centered k‐mesh of 21 × 21 × 1 and 5 × 5 × 1 were used for a single unit‐cell and a 4 × 4 × 1 supercell, respectively. The Tkatchenko‐Scheffler method was applied to cover the Van der Waals interaction between the adjacent layers. All structures were fully relaxed until the largest Hellmann‐Feynman force on each atom converged to values smaller than 0.01 eV per Å.

### Device Fabrication

For doped array devices, multilayer WSe_2_ film grew by CVD onto 300‐nm thick SiO_2_/p^++^ Si substrate (as gate dielectric/back‐gate). and then WSe_2_ film was patterned into rectangle stripes by using traditional photolithography and CF_4_ etching. First, the poly (methyl methacrylate) (PMMA A8, Mircochem Inc.) layer was spin‐coated on the substrate with a speed of 2500 r.p.m. for 30 s, then the array devices were moved to the preheated hotplate at 150 °C for 3 min to remove residual organic solvents. Next, source/drain electrodes were patterned by using standard e‐beam lithography and followed by thermal evaporation (50 nm Au) under vacuum (pressure ≈5 × 10^−4^ Pa). After the lift‐off process in acetone and isopropanol, the devices were immersed in 1.0 m LiPF_6_ solution with a 1:1 mixture of ethylene carbonate and diethyl carbonate solvents for 5 h in glove box with nitrogen environment. Finally, the samples were rinsed with ethanol and deionized water to remove residual LiPF_6_. In addition, the other devices were prepared by mechanical exfoliation. For the KPFM test, a WSe_2_ flake can be divided into two parts, one half was protected by a photoresist before being immersed in the LiPF_6_ solution and the other half was deliberately exposed and was immersed in the LiPF_6_ solution. The photoresist on the sample surface is removed with NMP and isopropyl alcohol. For fabricating logic inverter, PMOS device was obtained through the codoping strategy, while NMOS branch was protected by photoresist and achieved using evaporated Bi/Au (10/40 nm) as source/drain contact.

### Materials and Device Characterization

The XPS spectra were obtained using X‐ray photoelectron spectrometer (ESCALAB XI+). Raman and PL spectra were measured using a Raman spectrometer (LabRam HR Evolution) with 532 nm laser excitation. The thickness of WSe_2_ and KPFM measurements were conducted by atomic force microscopy (Cypher S). The FTIR spectra were measured using a Fourier infrared spectrometer (Vertex 80, Bruker). Integrated iDPC‐STEM and HAADF‐STEM were performed on the aberration‐corrected transmission electron microscopy (Spectra 300). The EELS profile confirmed the composition of the doped elements. XRD measurements (Bruker D8) were carried out on high‐resolution diffractometer. All electrical measurements of devices were exclusively conducted using a semiconductor parameter analyzer (B1500A, Keithley) and Lakeshore PS‐100 cryogenic probe station.

## Conflict of Interest

The authors declare no conflict of interest.

## Supporting information



Supporting Information

## Data Availability

The data that support the findings of this study are available from the corresponding author upon reasonable request.
