# Supplementary material for: Intralayer/Interlayer Codoping Stabilizes Polarity Modulation in 2D Semiconductors for Scalable Electronics
Source: Adv Sci (Weinh). 2024 Oct 24;11(48):2408634. doi: 10.1002/advs.202408634 (PMC11672281; doi:10.1002/advs.202408634)
Supplement: Supplementary file 1 — Supporting Information [file ADVS-11-2408634-s001.docx]

Supporting Information

Intralayer/interlayer codoping stabilizes polarity modulation in 2D semiconductors for scalable electronics

*Guitian Qiu, Lingan Kong, Mengjiao Han, Qian Zhang,* Majeed Ur Rehman, Jianxian Yi, Lede Xian, Xiankai Lin, Aumber Abbas, Jiwei Chen, Yingjie Luo, Wenbo Li, Zhongchao Wei, Hongyun Meng,* Xiuliang Ma, and Qijie Liang**


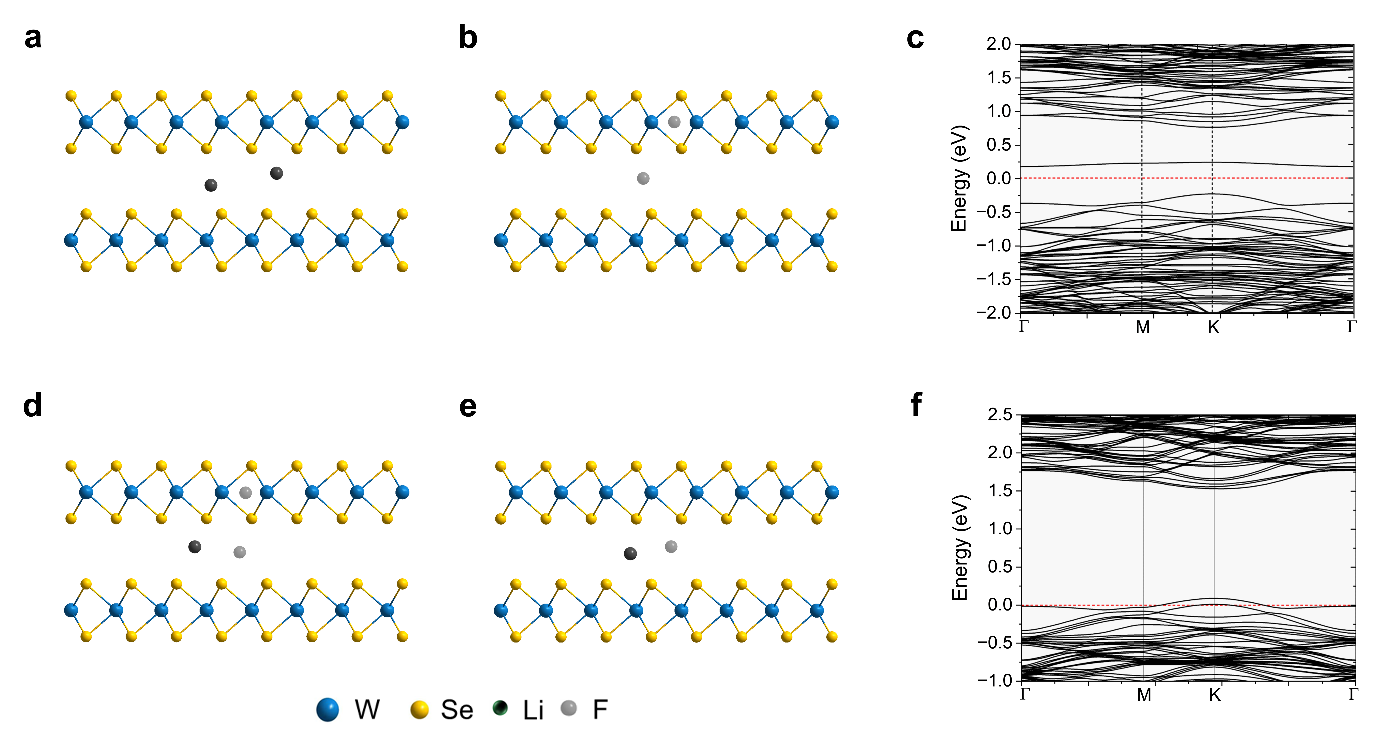


**Figure S1.** Structure diagram of (a) Li-doped WSe_2_ and (b) F-doped WSe_2_. (c) Energy band structure of F-doped WSe_2_. (d) Structure diagram of the intralayer/interlayer codoped WSe_2_. (e) Structure diagram of interlayer codoped WSe_2_. (f) Energy band structure of the interlayer codoped WSe_2_.


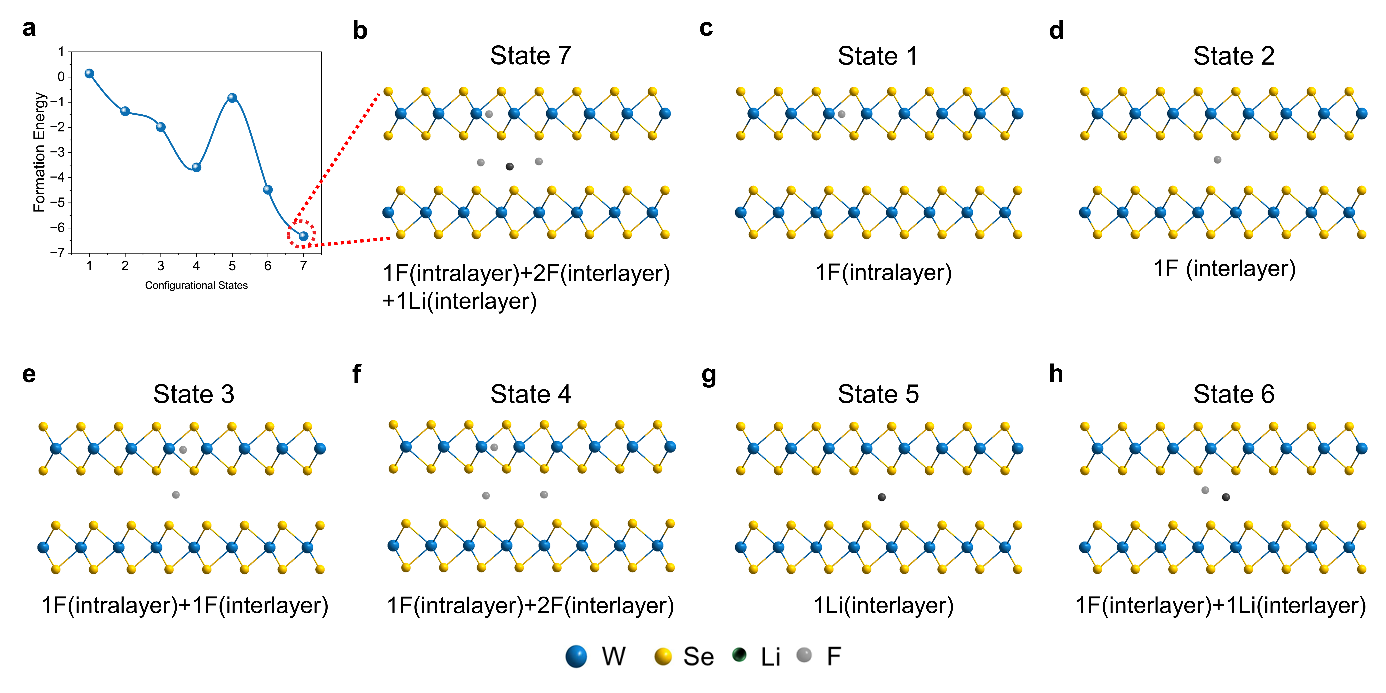


**Figure S2.** (a) Comparison of formation energies in different doping states. (b-h) Structural diagrams of F and Li ions in different doping states.

To assess the internal stability of dopants under different conditions, we calculated the formation energy ($E_{f}$) using the following formula:

$$E_{f}=E_{\text{doped}\text{ }}+E_{\text{clean}\text{ }}-n_{\text{Li}}\mu_{\text{Li}}-n_{F}\mu_{F}$$

where $E_{\text{doped}\text{ }}$and $E_{\text{clean}\text{ }}$represent the total energy of doped and clean (or undoped) WSe_2_ bilayer supercell, respectively. $n_{\text{Li}}$and$n_{F}$ are the numbers of Li and F ions doped in the WSe_2_ bilayer. $\mu_{\text{Li}}$and$\mu_{F}$ represent chemical potential of Li and F. We conducted a comprehensive analysis of F and Li ions doping in WSe_2_, as shown in Figure S2. For state 1, we first calculated that the presence of only 1F in the intralayer results in a positive formation energy. Consequently, the doped system exhibits maximum formation energy and is deemed highly unstable. When there is only 1F in the interlayer, although the formation energy drops to a negative value, it is still relatively large, so the doped system is not stable. Then, we analyzed the doped system with F ions both within the intralayer and interlayer, and found that the formation energy further decreased. Moreover, the formation energy was lower when there were 2F in the interlayer, and the system was more stable. Subsequently, we conducted an analysis of the doped system in the presence of only Li ion, and observed a significant increase in its formation energy, approaching zero. Furthermore, it was found that the doped system exhibited instability. Finally, we analyzed the states of F and Li ions codoped. The results show that the formation energy is lower in both states, and there is only F ion within the intralayer, while F and Li ions coexist between the interlayer to obtain the lowest formation energy and the most stable doped system. By comparing the formation energies of different states, we further confirm the high stability of the intralayer and interlayer codoping system we have adopted.


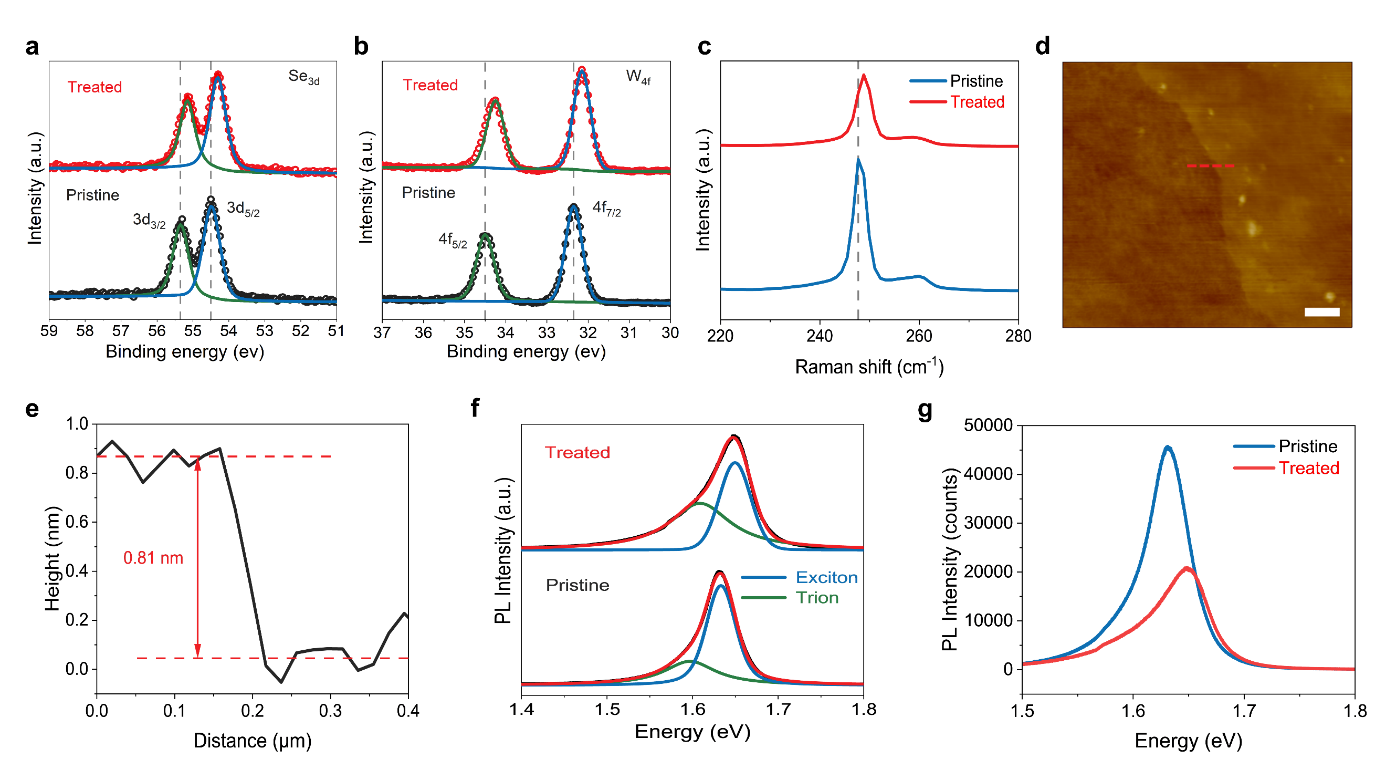


**Figure S3.** (a, b) XPS spectra (Se_3d_ and W_4f_) of the pristine and doped WSe_2_. (c) Raman spectra of pristine and doped WSe_2_. (d) The AFM image of the sample was analyzed by PL. Scale bar: 1 μm. (e) The height profile of the WSe_2_. (f) PL spectra of pristine and doped WSe_2_. (g) Comparison of PL intensity between pristine and doped WSe_2_.


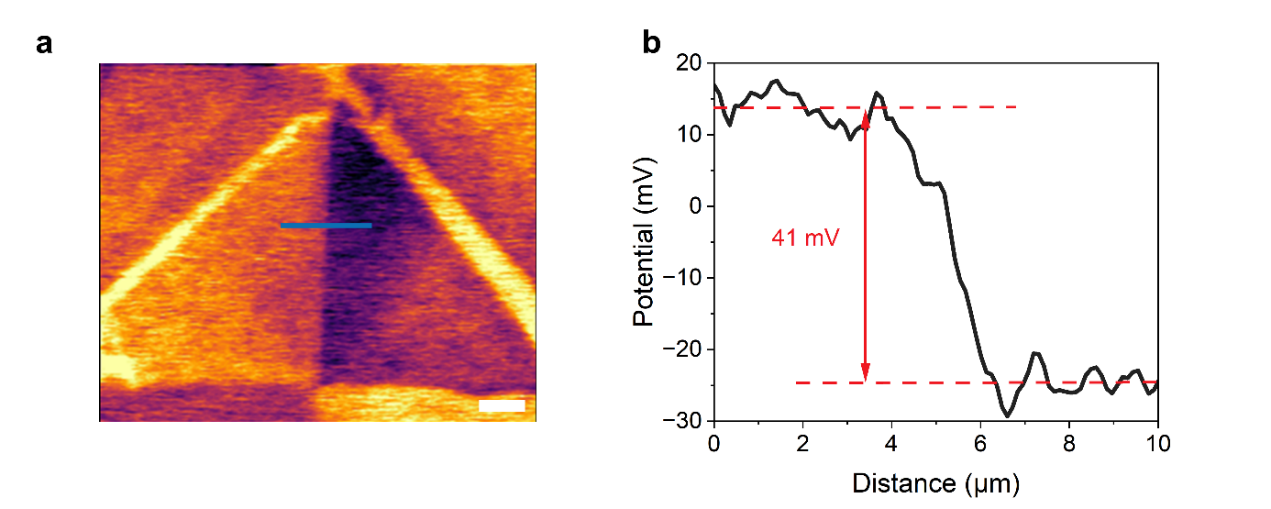


**Figure S4.** (a) The comparison of KPFM mapping of the WSe_2_ flake. Scale bar: 6 μm. (b) The corresponding surface potential difference of WSe_2_ is marked by the blue line in (a).


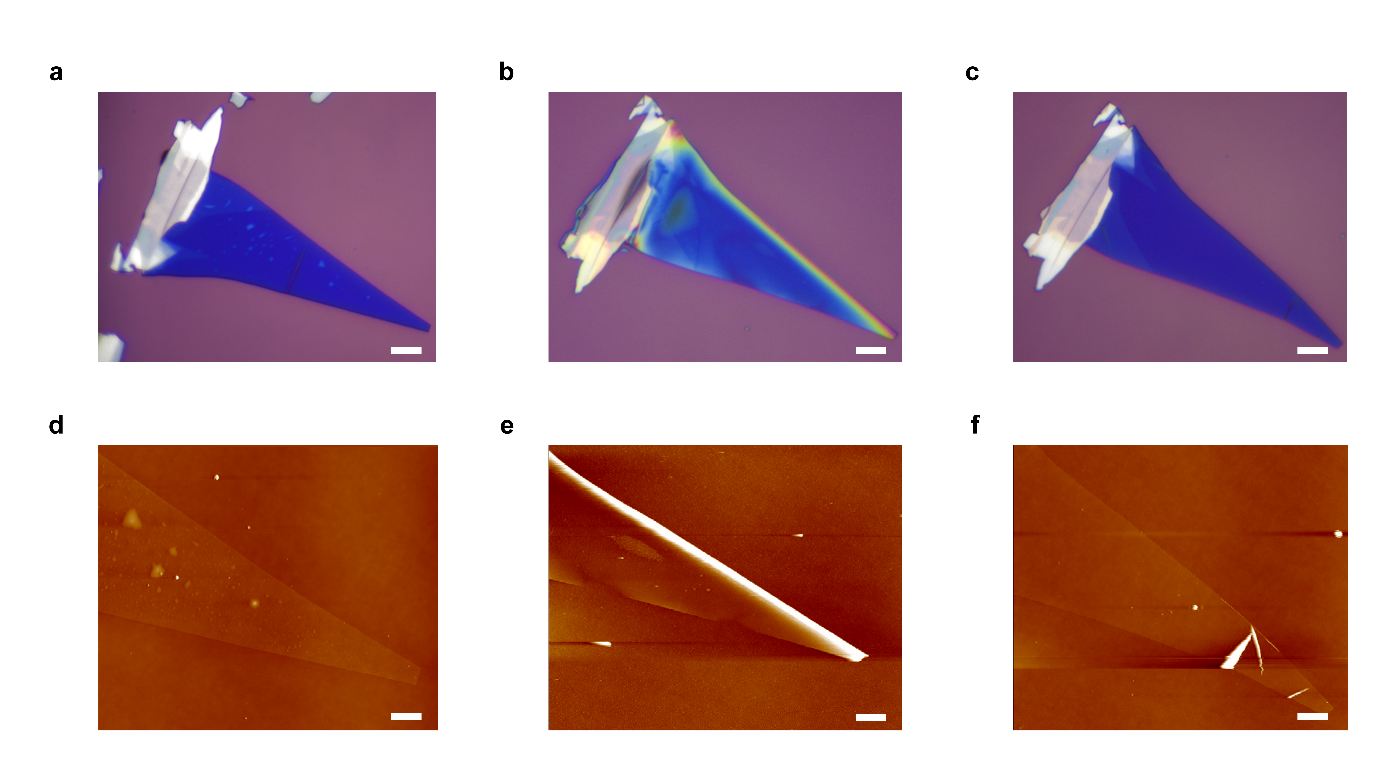


**Figure S5.** (a-c) Optical images of pristine WSe_2_, after 40 seconds, and 20 minutes of the removal of photoresist. Scale bar: 10 μm. (d-f) Corresponding AFM images of (a)-(c). Scale bar: 6 μm.


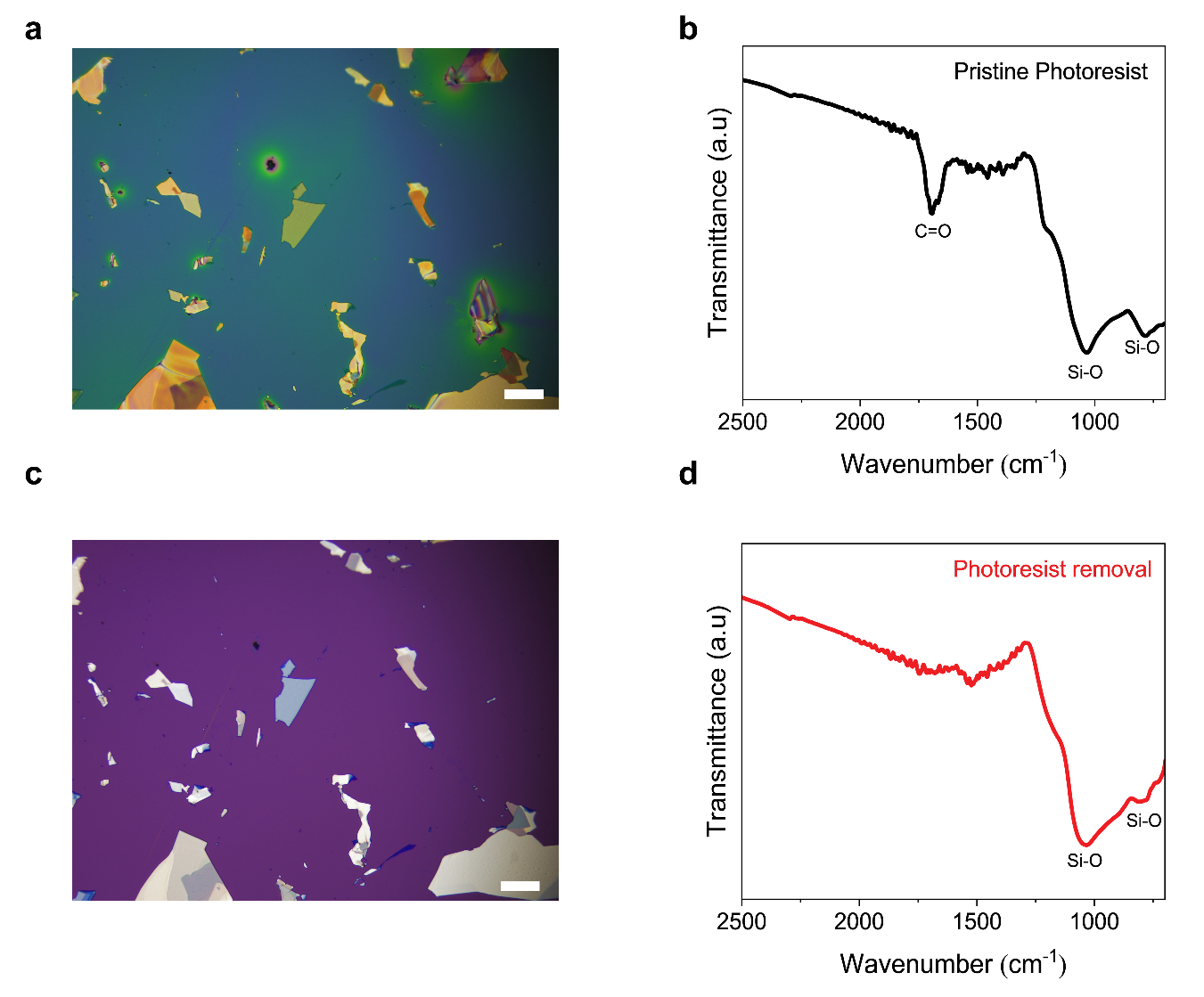


**Figure S6.** (a) Optical image after spin-coating photoresist. Scale bar: 50 μm. (b) FTIR spectrum of the pristine photoresist. (c) Optical image after removing photoresist. Scale bar: 50 μm. (d) FTIR spectrum of the photoresist removal.


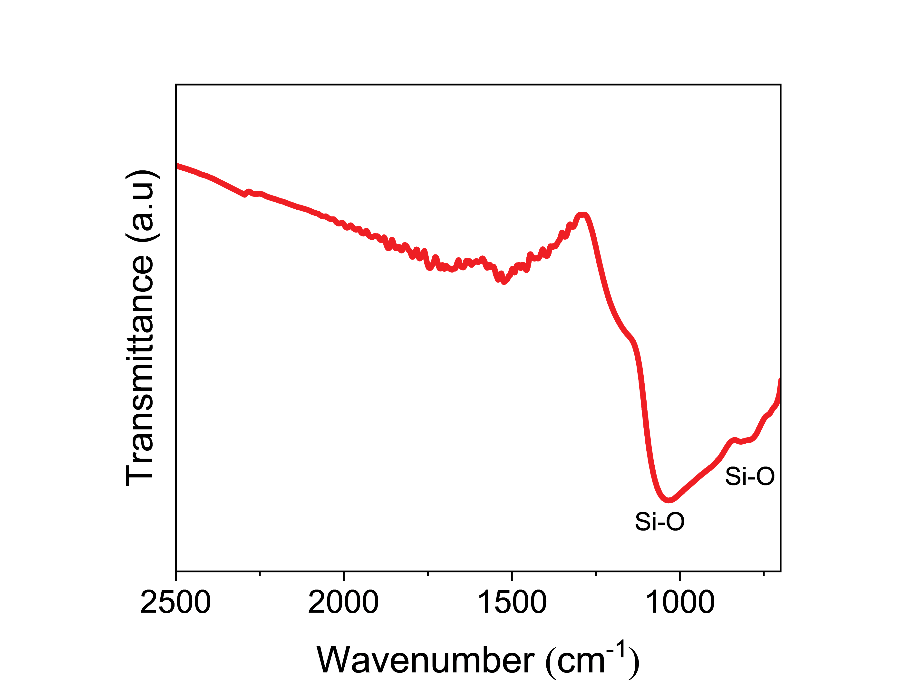


**Figure S7.** FTIR spectrum of the KPFM test sample.

To eliminate the influence of photoresist on the KPFM test results, we conducted a comparative experiment on the removal time of photoresist. In Figure S5, the results revealed that residual photoresist remained on WSe_2_ surface after 40 seconds of treatment. However, after 20 minutes of treatment, residue of photoresist on WSe_2_ surface has completely removed, which verified through optical microscopy and atomic force microscopy. Additionally, we compared that the changes in Fourier transform infrared (FTIR) spectra before and after the photoresist was removed. In Figure S6a, b, the sample with photoresist exhibits a pronounced absorption peak at 1693 cm^-1^, which is attributed to the stretching vibration of the C=O bond. Conversely, the absorption peak clearly disappeared after the photoresist removal, as shown in Figure S6c, d. We conducted FTIR spectroscopy on the sample (20 minutes of removal of photoresist) used for KPFM testing shown in Figure S4. The result indicated the absence of absorption peaks associated with the photoresist on the sample surface (Figure S7). Therefore, the WSe_2_ covered with photoresist part does not affect the KPFM test results, and the change in surface potential is due to the doping effect in the LiPF_6_ solution.


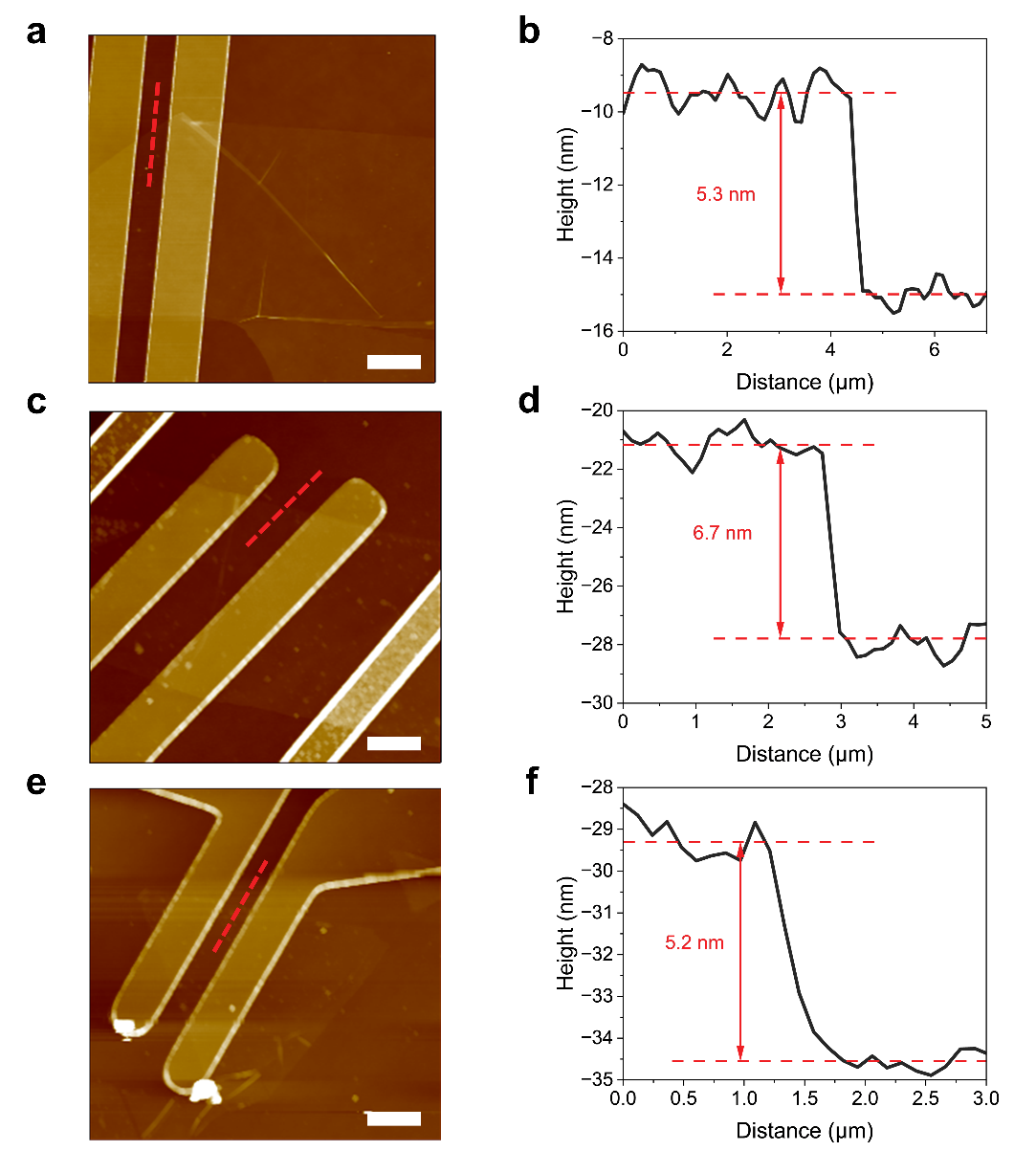


**Figure S8.** (a-f) AFM images and height profiles of the three WSe_2_ transistors randomly selected from 60 samples. Scale bar: 6 μm.

**
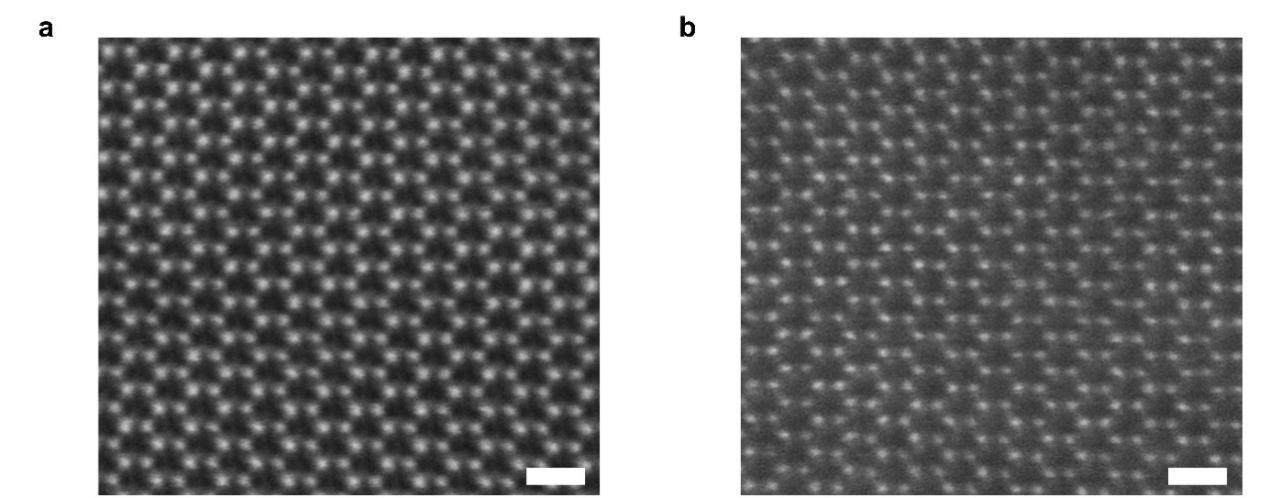
**

**Figure S9. (**a) HAADF-STEM image of the pristine WSe_2_ flake. (b) HAADF-STEM image of the codoped WSe_2_ flake. Scale bar: 1 nm.

**
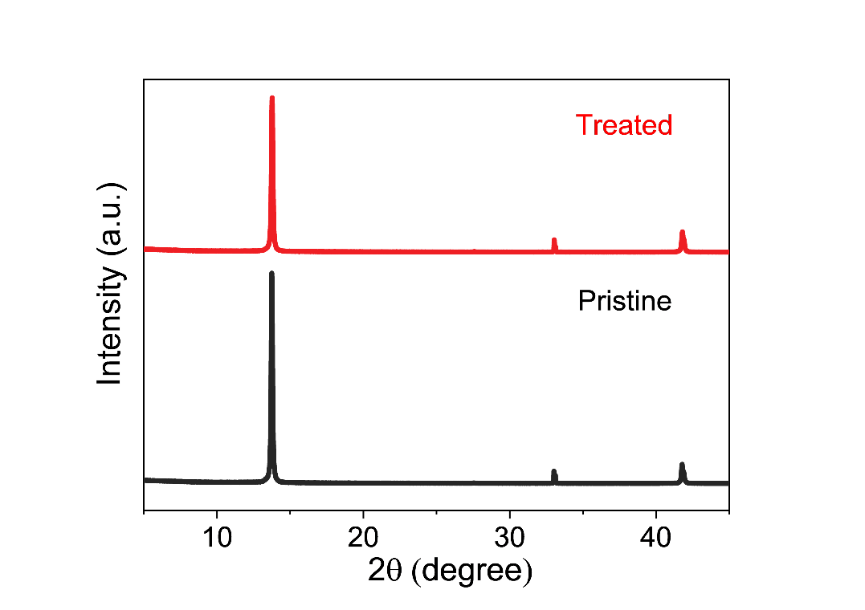
**

**Figure S10.** XRD spectra for the pristine and doped WSe_2_.

**
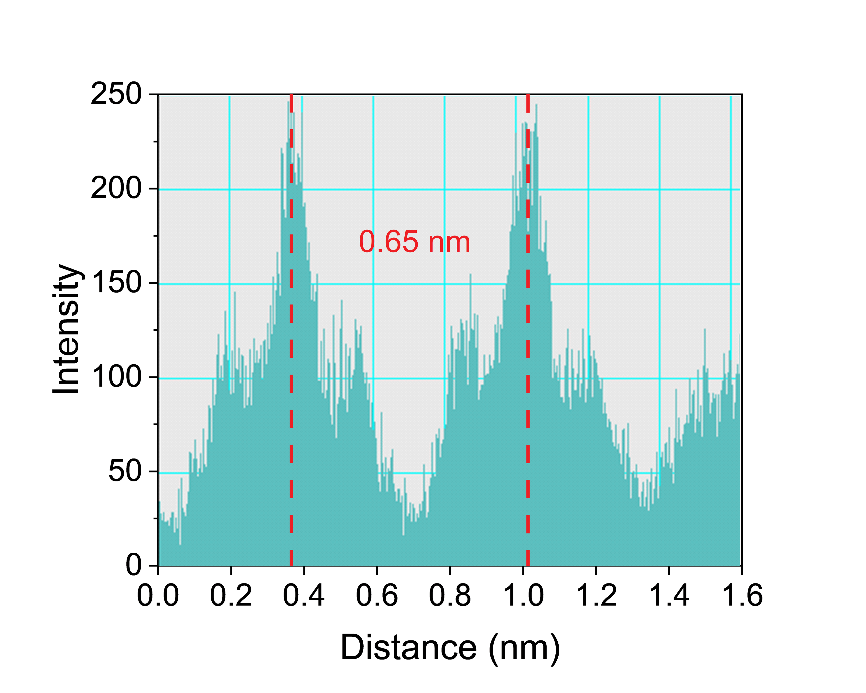
**

**Figure S11.** The layer spacing in the doped WSe_2_.

**
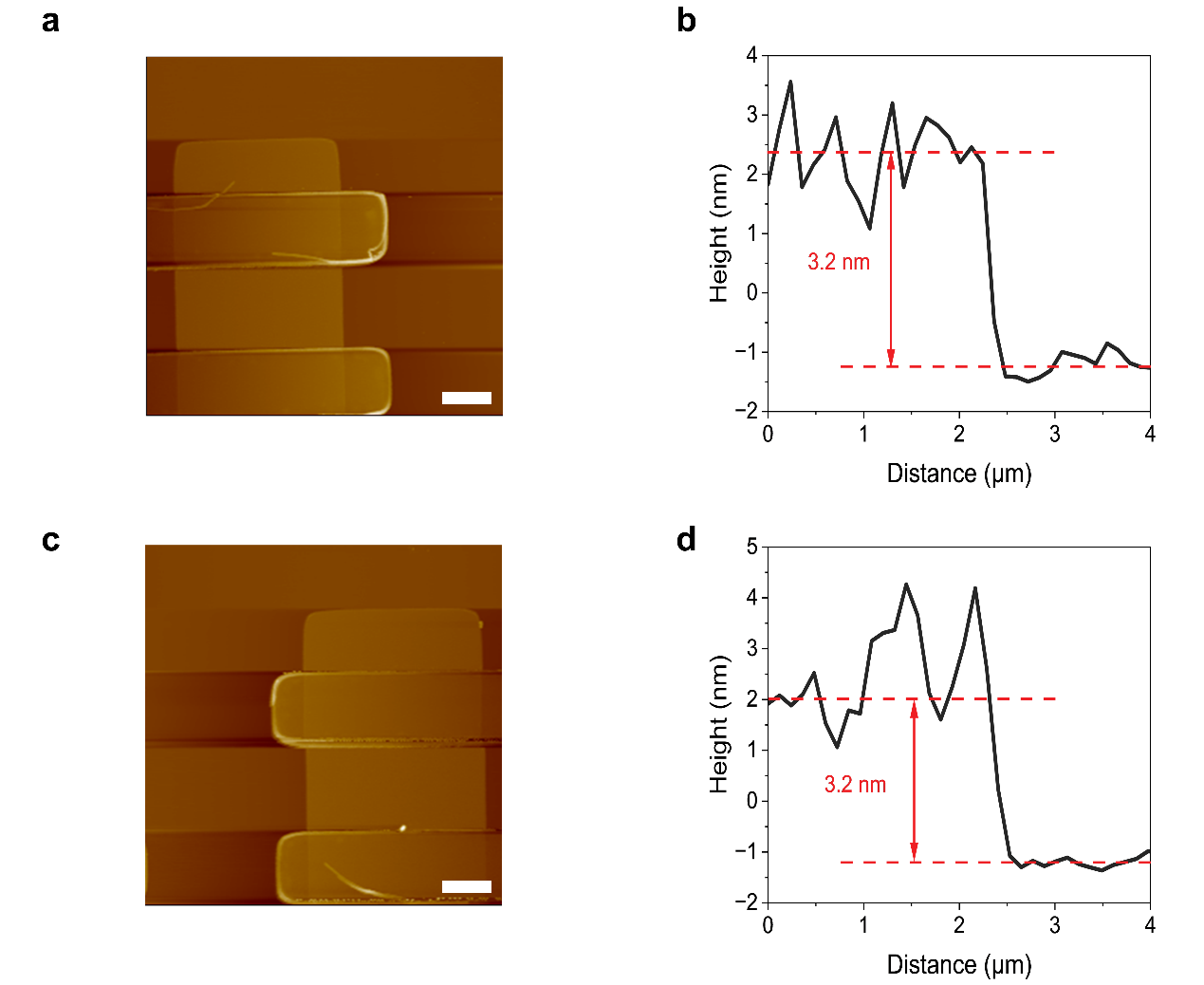
**

**Figure S12.** (a-d) AFM images and height profiles of WSe_2_ device arrays. Scale bar: 6 μm.


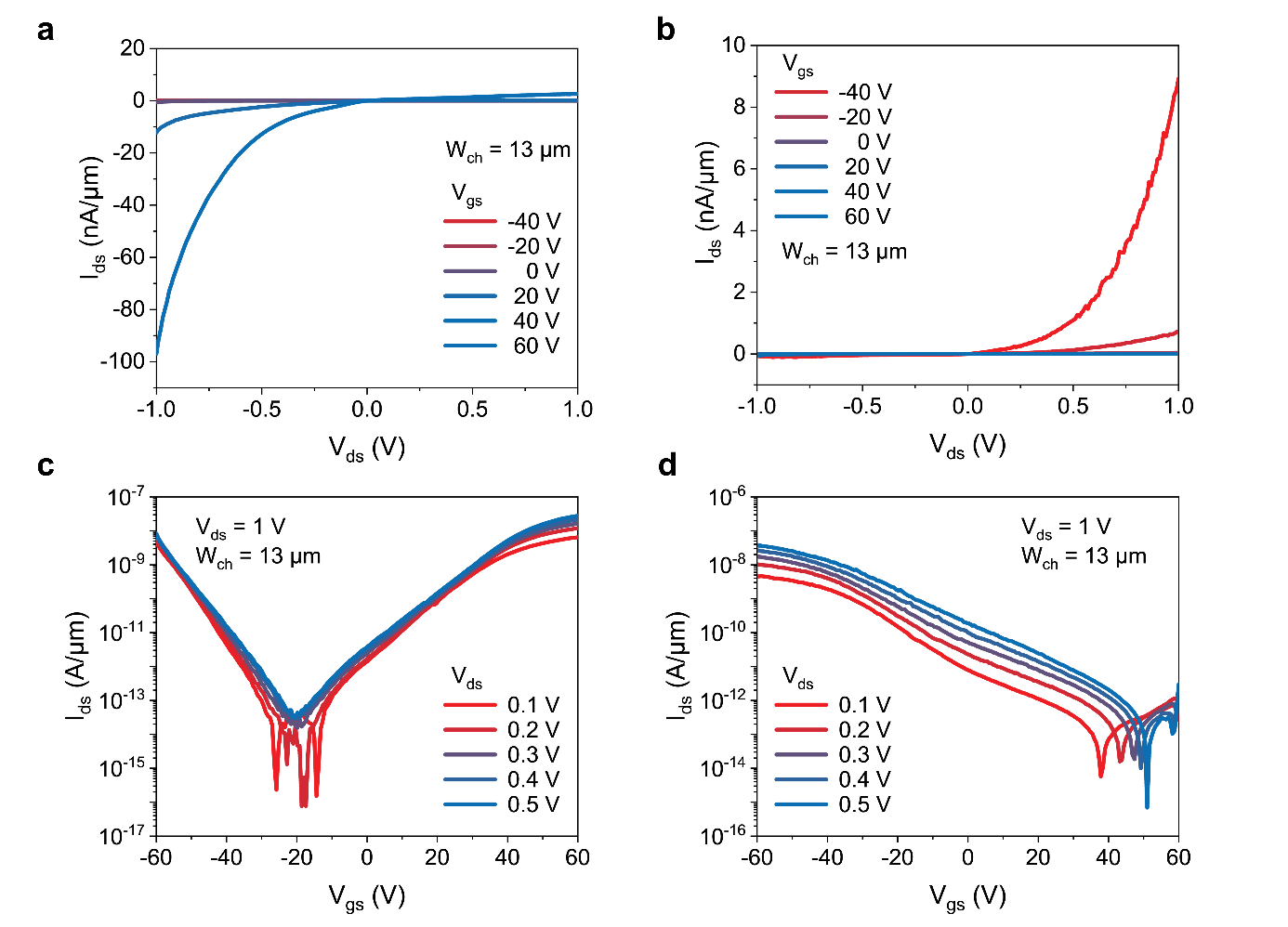


**Figure S13.** (a, b) Output curves of the pristine and doped WSe_2_ transistor with different V_gs_ from -40 to 60 V. (c, d) Transfer curves of the pristine and doped WSe_2_ transistor with varied V_ds_ from 0.1 to 0.5 V.


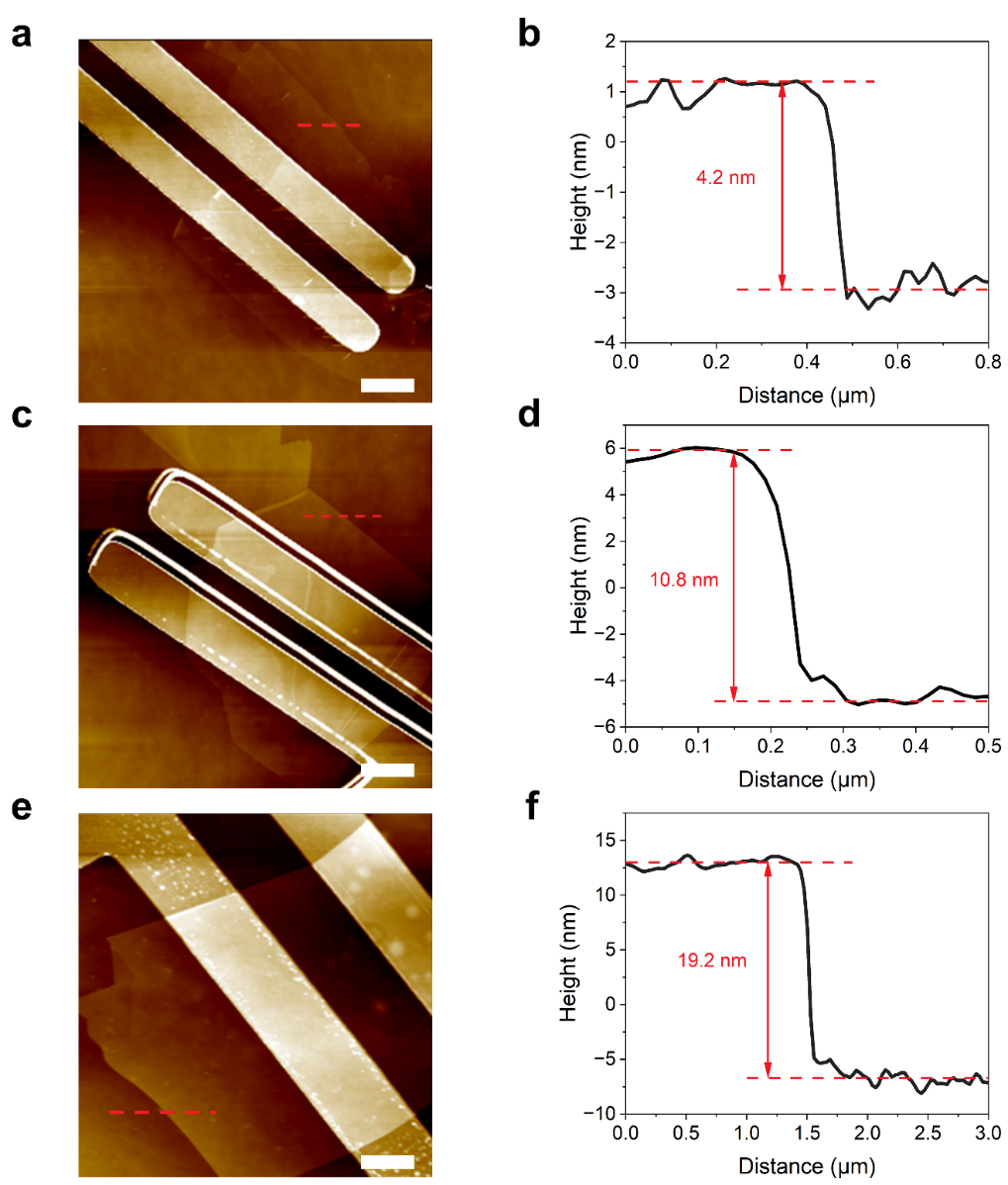


**Figure S14.** (a-f) AFM images and height profiles of the WSe_2_ transistors with thickness of 4.2 nm,10.8 nm and 19.2 nm, respectively. Scale bar: 6 μm.


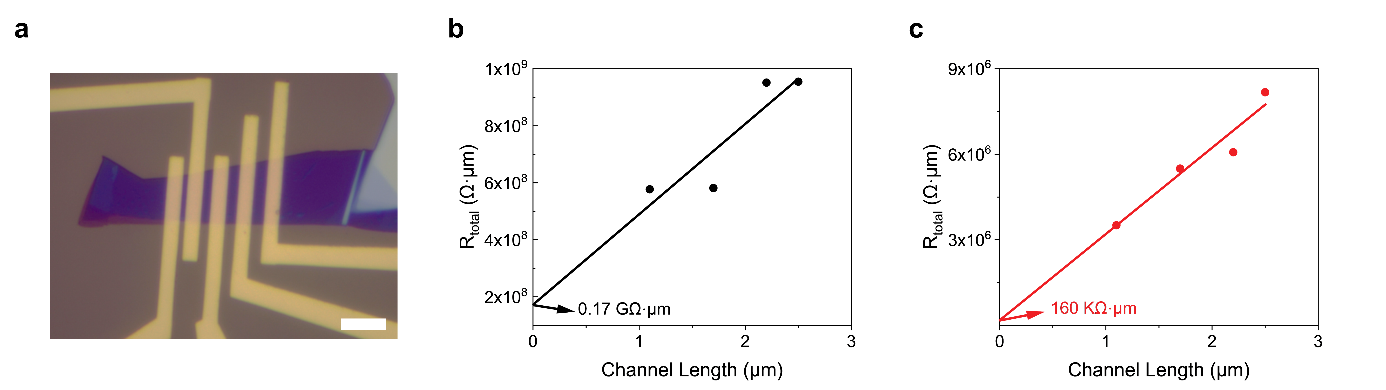


**Figure S15.** (a) Optical image of the fabricated devices with transmission line method configuration. Scale bar: 10 μm. (b, c) Extracted changes of total resistance without (black solid circles) and with (red solid circles) LiPF_6_ treatment.

The enhancement of hole mobility in these devices is closely related to the metal-semiconductor contact. We use the transfer line method (TLM) to extract the contact resistance (R_C_) before and after codoping, as shown in Figure S15a. According to R_total_ = R_channel_ + 2R_C_, where R_total_ is the measured total resistance between two metal electrodes, and R_channel_ is the channel resistance. Then the 2R_C_ can be extracted from the intercept of the linear fitting of R_total_ with respect to channel length. In Figure S15b, c, 2R_C_ is estimated to be 0.17 GΩ·μm and 160 kΩ·μm at on-state (V_g_ = -60 V) for WSe_2_ FET before and after LiPF_6_ codoping, respectively, indicating a significant reduction of R_C_ between WSe_2_ and Au metal electrode by three orders of magnitude via codoping.


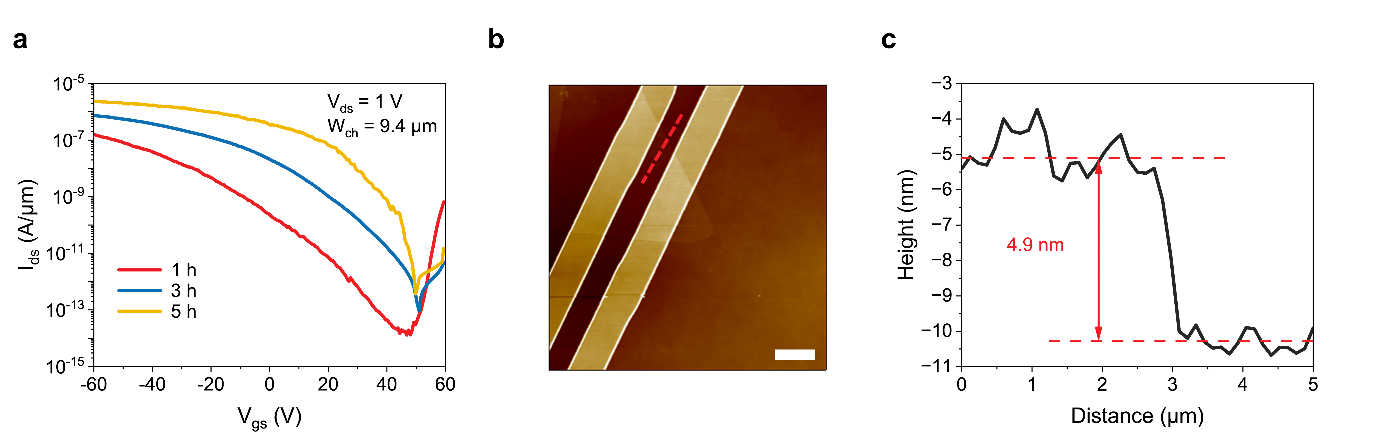


**Figure S16.** (a) Transfer curves of the WSe_2_ transistor with different doping times. (b, c) AFM image and height profile of WSe_2_ transistor with different doping times. Scale bar: 6 μm.


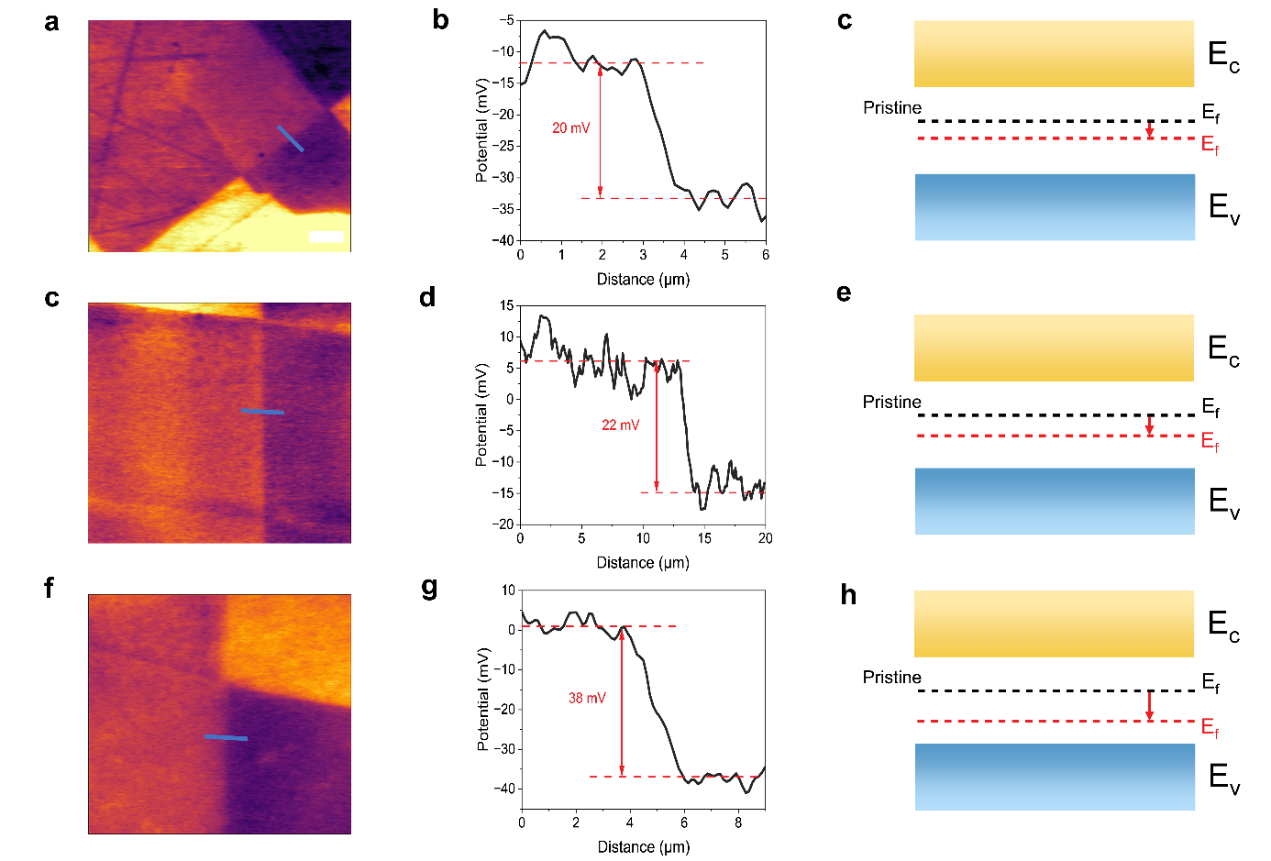


**Figure S17.** (a-f) KPFM mapping and corresponding surface potential (marked by the blue line) and schematic of the change of the energy band structure of WSe_2_ flakes with different immersion times in LiPF_6_ solution. Scale bar: 6 μm.


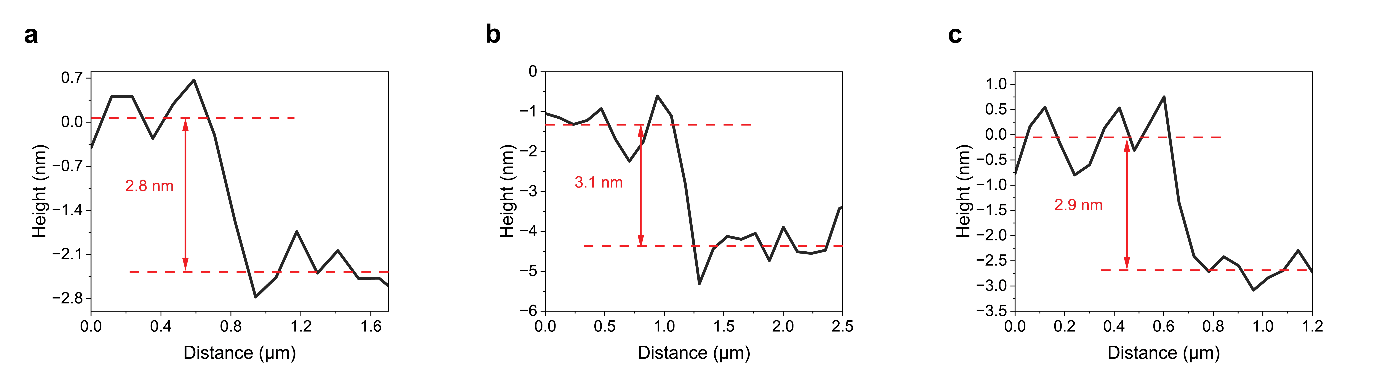


**Figure S18.** (a-c) The height profiles of WSe_2_ flakes after immersion in LiPF_6_ solution for 30 minutes, 1 hour, and 3 hours, respectively.


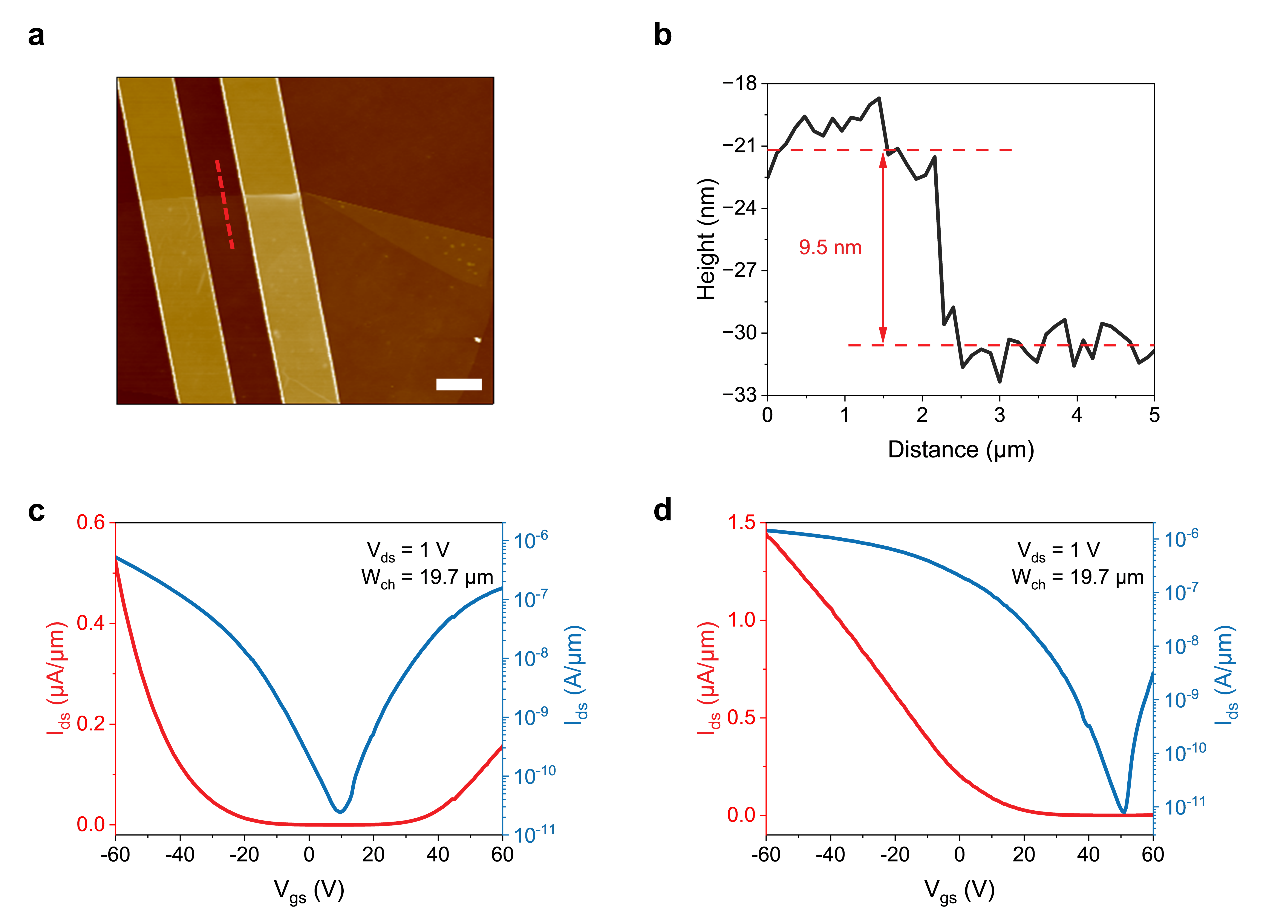


**Figure S19.** (a, b) AFM image and height profile of MoTe_2_ transistor. Scale bar: 6 μm. (c, d) I_ds_-V_gs_ transfer characteristics of the pristine and codoped MoTe_2_ transistor.

**
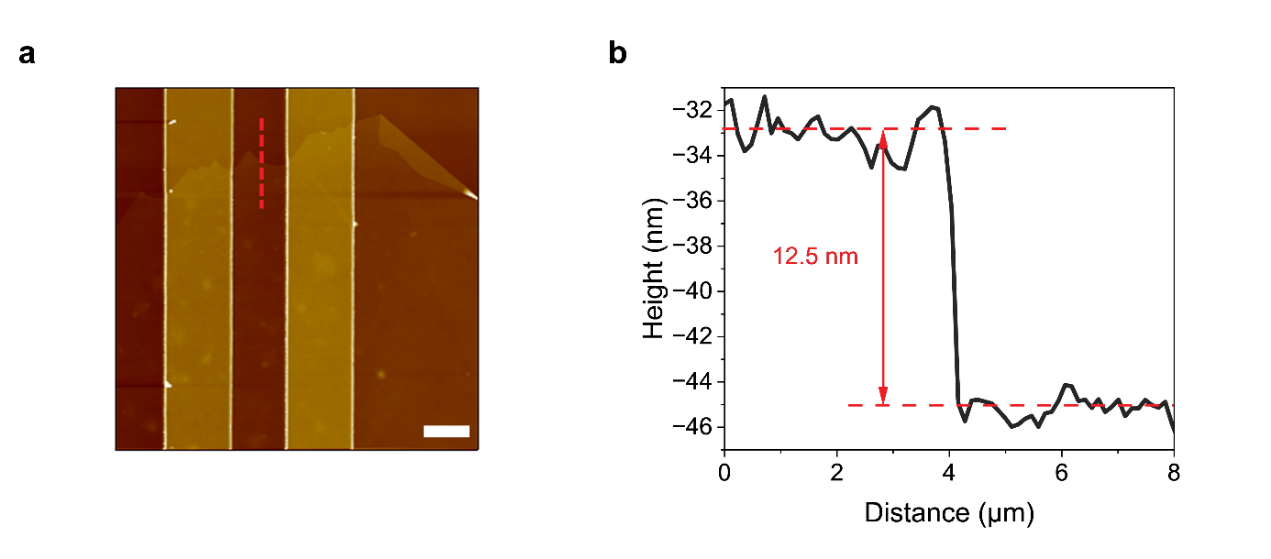
**

**Figure S20.** (a, b) AFM image and height profile of WSe_2_ transistors used for temperature stability testing. Scale bar: 6 μm.


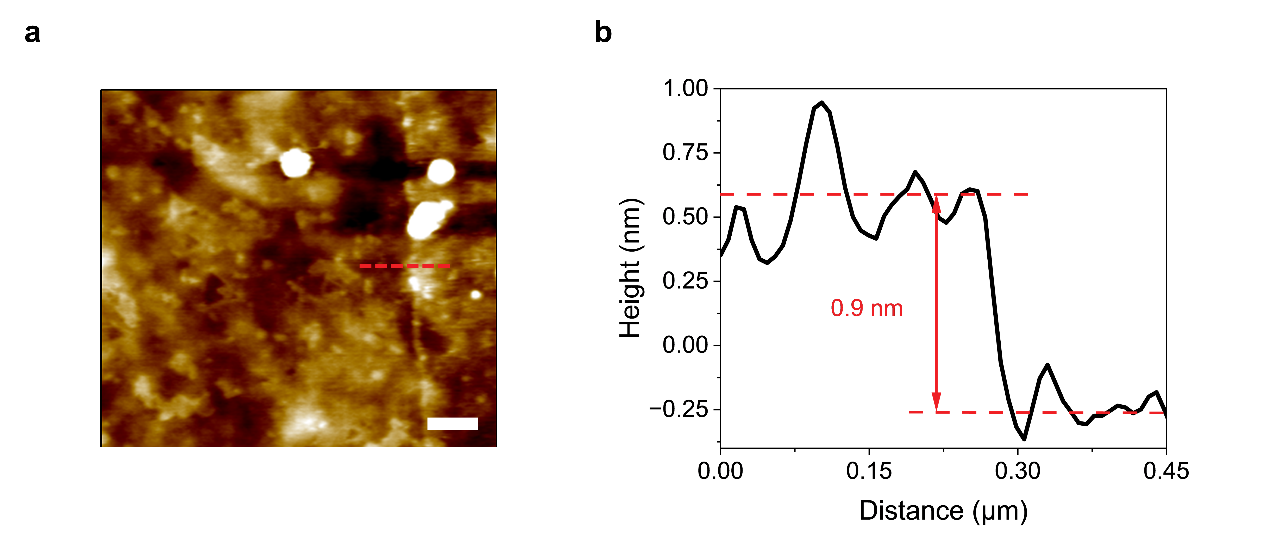


**Figure S21.** (a, b) AFM image and height profile of the codoped WSe_2_ transistors with H_2_ plasma treatment. Scale bar: 0.4 μm.


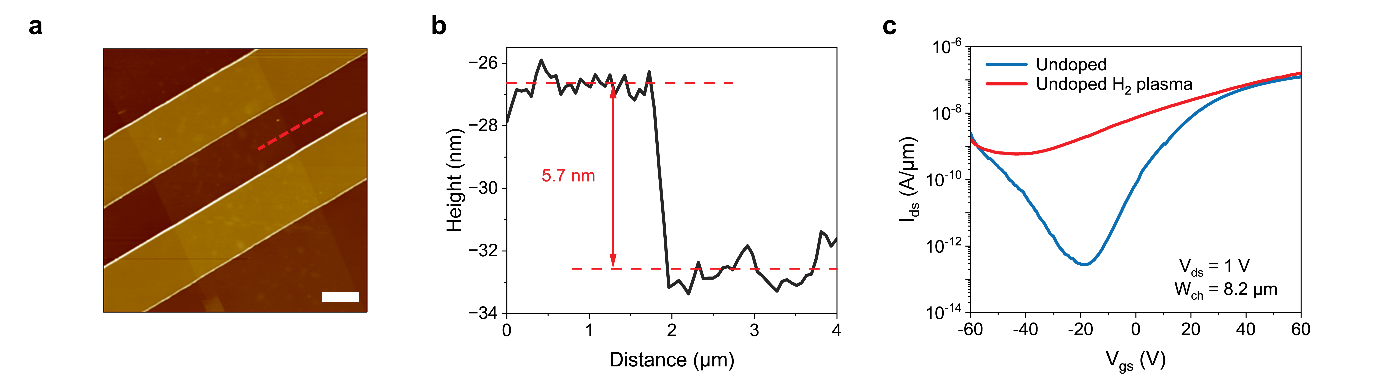


**Figure S22.** (a, b) AFM image and height profile of WSe_2_ transistors used for H_2_ plasma treatment. Scale bar: 6 μm. (c) Transfer characteristics of the undoped WSe_2_ transistor before and after H_2_ plasma treatment.


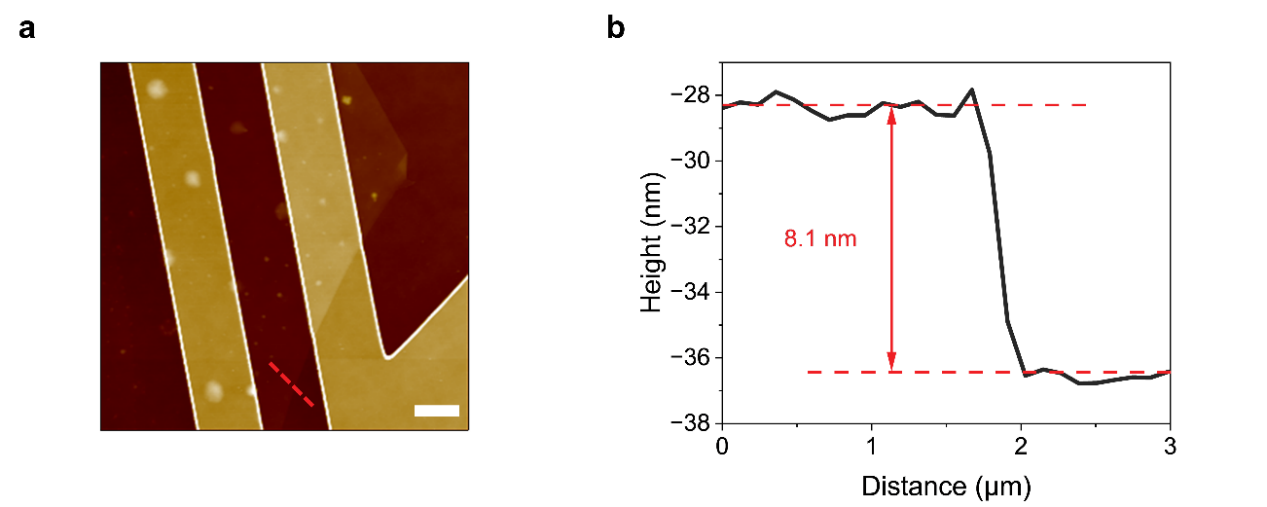


**Figure S23.** (a, b) AFM image and height profile of WSe_2_ transistors used for time stability testing. Scale bar: 6 μm.


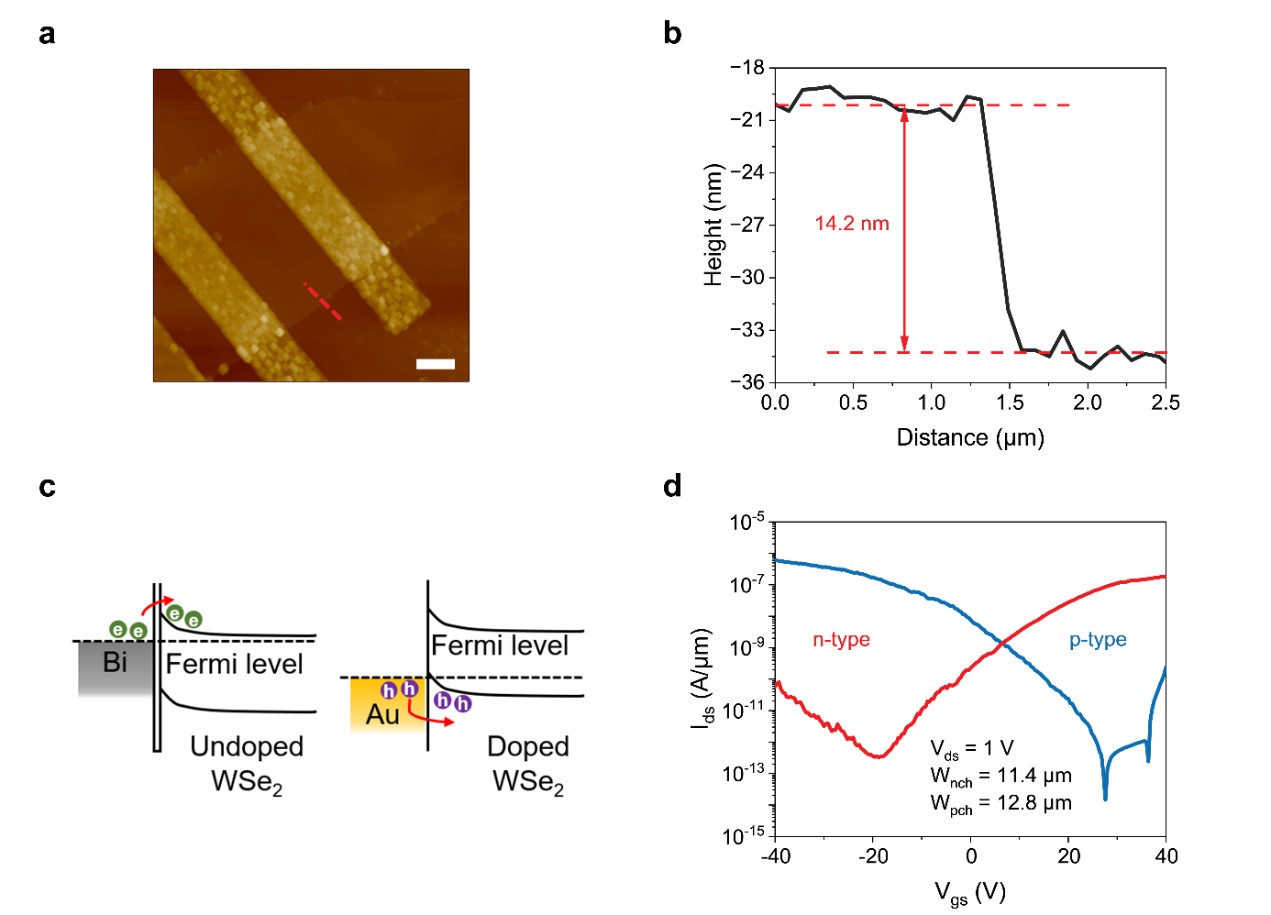


**Figure S24.** (a, b) AFM image and height profile of the inverter. Scale bar: 6 μm. (c) Band diagram schematics of metal Bi and Au respectively contact to undoped and doped multilayer WSe_2_. (d) Transfer curves for evaporated Bi-contacted device (n-type) and codoped device (p-type).


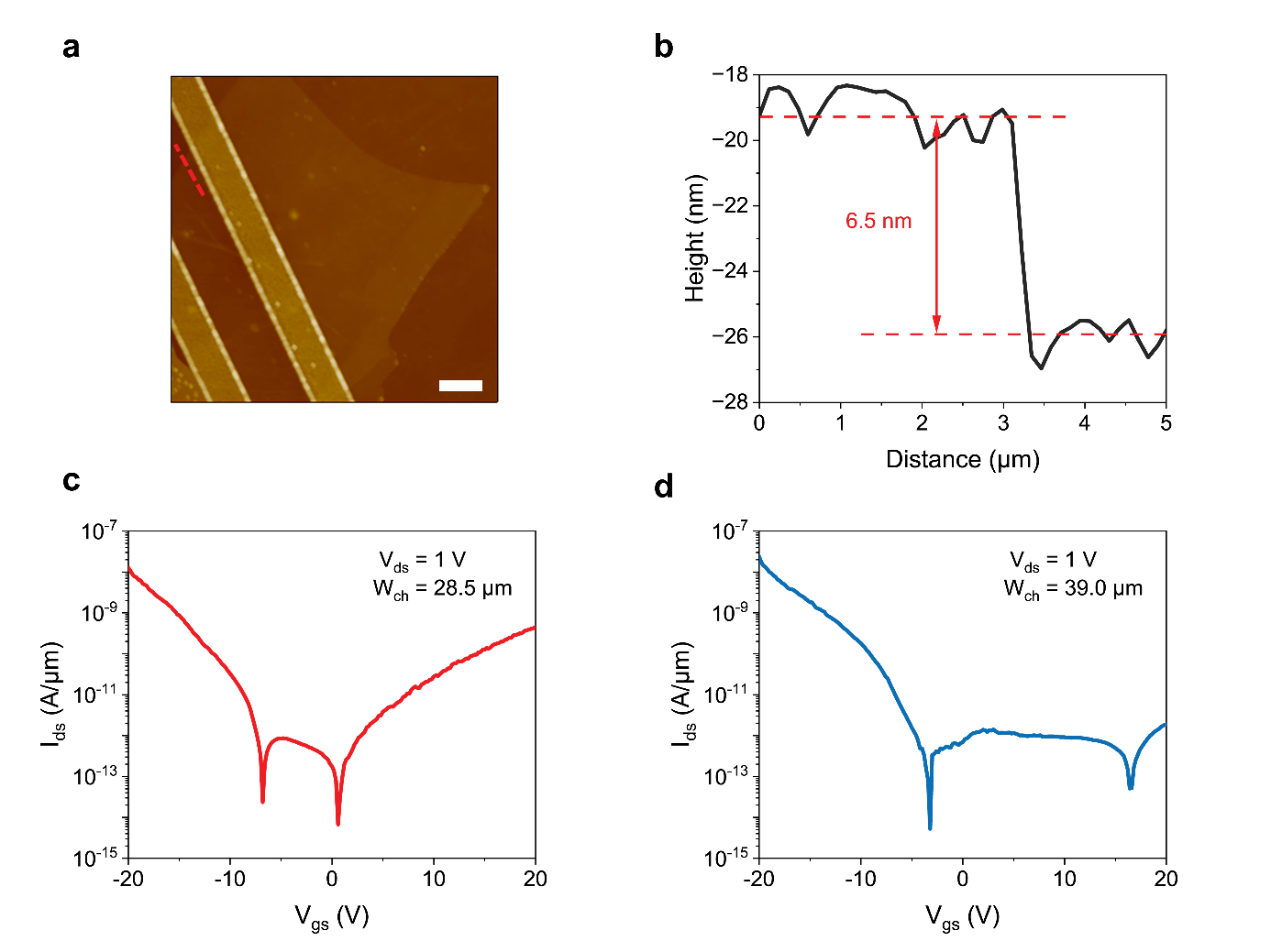


**Figure S25.** (a, b) AFM image and height profile of NOR and XNOR logic circuits. Scale bar: 6 μm. (c, d) Transfer characteristics of ambipolar (20 nm h-BN/SiO_2_ substrate) and p-type (SiO_2_ substrate) codoped WSe_2_.
